# Exploring an EM-algorithm for banded regression in computational neuroscience

**DOI:** 10.1162/imag_a_00155

**Published:** 2024-05-20

**Authors:** Søren A. Fuglsang, Kristoffer H. Madsen, Oula Puonti, Hartwig R. Siebner, Jens Hjortkjær

**Affiliations:** Danish Research Centre for Magnetic Resonance, Centre for Functional and Diagnostic Imaging and Research, Copenhagen University Hospital—Amager and Hvidovre, Copenhagen, Denmark; Hearing Systems Section, Department of Health Technology, Technical University of Denmark, Kgs. Lyngby, Denmark; Department of Applied Mathematics and Computer Science, Technical University of Denmark, Kgs. Lyngby, Denmark; Martinos Center for Biomedical Imaging, Massachusetts General Hospital and Harvard Medical School, Boston, MA, United States; Department of Neurology, Copenhagen University Hospital Bispebjerg, Copenhagen, Denmark; Institute for Clinical Medicine, Faculty of Health and Medical Sciences, University of Copenhagen, Copenhagen, Denmark

**Keywords:** encoding, decoding, EEG, fMRI, regularization

## Abstract

Regression is a principal tool for relating brain responses to stimuli or tasks in computational neuroscience. This often involves fitting linear models with predictors that can be divided into groups, such as distinct stimulus feature subsets in encoding models or features of different neural response channels in decoding models. When fitting such models, it can be relevant to allow differential shrinkage of the different groups of regression weights. Here, we explore a framework that allows for straightforward definition and estimation of such models. We present an expectation-maximization algorithm for tuning hyperparameters that control shrinkage of groups of weights. We highlight properties, limitations, and potential use-cases of the model using simulated data. Next, we explore the model in the context of a BOLD fMRI encoding analysis and an EEG decoding analysis. Finally, we discuss cases where the model can be useful and scenarios where regularization procedures complicate model interpretation.

## 1 Introduction

Understanding how the brain responds to different stimuli or tasks is a key objective in systems neuroscience. In recent years, significant progress has been made in developing models to analyze neural responses to complex, naturalistic stimuli (Hamilton & Huth, 2020). One potent approach is encoding models that combine stimulus features to predict channel-wise neural responses from fMRI or electrophysiology through linear regression (Broderick et al., 2018;de Heer et al., 2017;Di Liberto et al., 2015;Dumoulin & Wandell, 2008;Kay et al., 2013;Kell et al., 2018). Regularization is often needed in such models to reduce the risk of over-fitting and improve model prediction on held-out data. However, multiple - potentially colinear - stimulus feature sets can complicate the assessment of the relative importance of the individual features. In this case, it can be relevant to allow for differential shrinkage of different groups of regression weights. For instance, neural responses to natural speech depend on both low-level acoustic features and high-level phonetic or semantic features (Broderick et al., 2018;Di Liberto et al., 2015;Huth et al., 2016;Jain & Huth, 2018). Such feature groups vary on different time scales, have different dimensionality, and some features may not even be reflected in the neural response.

Several regularization strategies could have relevance for this situation (David et al., 2007;Golub et al., 1999;Hoerl & Kennard, 1970;Massy, 1965;Park & Pillow, 2011;Sahani & Linden, 2002;Tibshirani, 1996;Tibshirani et al., 2005;Tikhonov & Arsenin, 1977;Zou & Hastie, 2005). Recently,Nunez-Elizalde et al. (2019)andla Tour et al. (2022)proposed to divide predictors in encoding analyses into separate feature “bands” (groups of predictors) and allow differential shrinkage of weights associated with these different bands. The authors proposed banded Ridge regression - also referred to as grouped Ridge regression (van de Wiel et al., 2021) - and showed that banded Ridge regression can yield higher out-of-sample predictive accuracy compared to standard Ridge regression in fMRI encoding analyses (la Tour et al., 2022;Nunez-Elizalde et al., 2019). Other methods that can incorporate grouping structure constraints and other priors (Boss et al., 2023;Friedman et al., 2010;Simon et al., 2013;van de Wiel et al., 2016;Xu & Ghosh, 2015;Yuan & Lin, 2006;Zhao et al., 2009) could also be relevant in encoding analyses with grouped features.

Similar regression problems arise in decoding models, that is, models that combine neural channels (voxels, electrodes) to predict a continuous stimulus feature or task feature (Naselaris et al., 2011;Wu et al., 2006). One pertinent example is decoding models for EEG or MEG responses to continuous stimuli, like speech or music (Di Liberto et al., 2021;Ding & Simon, 2012;O’Sullivan et al., 2014). Here, regularized regression can be used to identify multidimensional finite impulse response (FIR) filters that predict stimulus features by combining different electrode signals at different time lags and/or different frequency bands (de Cheveigné et al., 2018;Mesgarani & Chang, 2012;Mirkovic et al., 2015). As with encoding models, it can be useful to allow for differential shrinkage of groups of weights corresponding to groups of predictors.

Here, we explore an empirical Bayes framework (Efron & Morris, 1973,^1975^;Morris, 1983;Robbins, 1964) for estimating “banded”-type regression models. The framework is closely related to automatic relevance determination (Bishop, 2006;MacKay, 1994;Park & Pillow, 2011;Sahani & Linden, 2002;Tipping, 2001;Wipf & Nagarajan, 2007) and can be seen as an extension of work byTipping (2001). The framework can be used to formulate regularized regression estimators with different regularization properties. We use an expectation-maximization (EM) algorithm to tune hyperparameters that control variances of prior distributions. The model can be used to formulate priors that allow for differential shrinkage of groups of regression weights. The model can additionally be used to promote smoothness on groups of regression weights by encouraging correlation between related regression weights.

In this paper, we describe the modeling framework and provide simulations to illustrate the properties, limitations, and potential use-cases of the described algorithm. Additionally, we illustrate properties of the model by using it to fit stimulus-response models on two publicly available datasets (Broderick et al., 2018;Nakai et al., 2022) and compare these model fits with the widely used Ridge estimator (Hoerl & Kennard, 1970). In the first dataset, we consider encoding models that use a set of audio features to predict BOLD fMRI responses to music (Nakai et al., 2022). In the second dataset, we fit decoding models that use features of multichannel time-lagged EEG data to predict an audio stimulus feature during a speech listening task (Broderick et al., 2018). Finally, we describe how simplifying model assumptions can allow for an efficient estimation in regression problems with multiple target variables. Software implementations of the model in both MATLAB and Python are available athttps://github.com/safugl/embanded.

## 2 Methods

### 2.1 Regression model

Encoding and decoding analyses often involve linear regression models in the following form:

y=Xw+ϵ, (1)

whereϵis a zero-mean Gaussian variable.

Here, encoding models would treat stimulus features or task features as predictors,X, and a (preprocessed) neural response as target variable,y. Decoding models treat features extracted from neural activity measurements as predictors,X, and a stimulus feature or task feature as target variable,y. The differences in direction of inference have large implications for model interpretation and model properties (Haufe et al., 2014;Naselaris et al., 2011) as well as how potential stimulus confounders or task confounders can be dealt with.

Despite their differences, such encoding and decoding models lead to regression problems that can be encompassed in a general formulation. Let$y\in {\text{R}}^{M\times 1}$denote a target variable whereMindicates the number of observations. Moreover, letXdenote a matrix withDpredictors such that$X\in {\text{R}}^{M\times D}$. Suppose thatXcan be divided into a set of meaningful groups such that$X=\left[F_{1},F_{2},...,F_{J}\right]$,j=1,2,​...,j,​...J. Here,$F_{j}$denotes a group of$D_{j}$predictors, that is,$F_{j}\in {\text{R}}^{M\times D_{j}}$. This group of predictors could, for example, be a stimulus feature subset in an encoding model, or response features of different voxels or electrodes in a decoding model. We thus consider a regression model in the following form (la Tour et al., 2022;Nunez-Elizalde et al., 2019):

$$y=F_{1}\beta_{1}+F_{2}\beta_{2}+...+F_{J}\beta_{J}+\epsilon ,$$ (2)

where$\beta_{j}$denotes regression weights for a given group of predictors,$F_{j}$. Further, letwdenote a column vector collecting all the corresponding weights$w={\left[\beta_{1}^{⊤},\beta_{2}^{⊤},...,\beta_{J}^{⊤}\right]}^{⊤}$, with$w\in {\text{R}}^{D\times 1}$. We will assume that input variables and target variables have been centered and that no intercept term is needed in the regression model (since the intercept typically carries no meaningful information and hence shrinkage is typically not desired). The conditional distribution of the target variableygiven a set of observed predictorsXand given the model with parameterswandνwill be assumed to follow a Gaussian distribution in the following form:

$$p(y|X,w,\nu )=N(y|Xw,\nu I_{M}),$$ (3)

where$N(y|Xw,\nu I_{M})$is used to indicate a multivariate Gaussian with meanXwand a diagonal covariance matrix$\nu I_{M}$of sizeM×M. A zero-mean Gaussian prior distribution will be placed over the weights:

p(w|​Λ,η,τ)=N(w|​0,Λ), (4)

whereΛis aD×Dblock-diagonal covariance matrix. Such a prior distribution over the weights can be used to “shrink” the estimated regression weights. Here, we specifically define thatΛhas a block structure defined as follows:

$$\begin{array}{ccc} \Lambda & \equiv \left[\begin{array}{ccc} \lambda_{1}I_{D_{1}} & \ldots & 0\\ \vdots & \ddots & \vdots \\ 0 & \ldots & \lambda_{J}I_{D_{j}} \end{array}\right]\left[\begin{array}{ccc} {\text{Ω}}_{1} & \ldots & 0\\ \vdots & \ddots & \vdots \\ 0 & \ldots & {\text{Ω}}_{J} \end{array}\right]. & \end{array}$$ (5)

By introducing such a prior distribution over the weights, it is now possible to allow for differential shrinkage of different groups of weights. We define that thej-th block inΛrelates to a single group of predictors,$F_{j}$, and that${\text{Ω}}_{j}$has size$D_{j}\times D_{j}$. The column indices inXunique to predictor group$F_{j}$will thus index rows and columns inΛfor that block. The terms${\text{Ω}}_{j}$,j=1,...,J, denote matrices that are assumed to be fixed*a priori*. For now, we will assume that they are identity matrices. The following parametrization (Matérn, 1960;Rasmussen & Williams, 2005) of these terms will later be used for encouraging local smoothness via a Matérn covariance function with order3​/​2and length scale$h_{j}$:

$${\text{Ω}}_{j}=\left(1+\frac{\sqrt{3}\left|k-i\right|}{h_{j}}\right)\text{exp}\left(-\frac{\sqrt{3}\left|k-i\right|}{h_{j}}\right),k=1,...,D_{j},i=1,...,D_{j}.$$ (6)

Note that rather than attempting to estimate$h_{j}$and impose hyperpriors on the$h_{j}$terms, we will fix these parameters*a priori*. We complete the model by placing Inverse-Gamma prior distributions over the$\lambda_{j}$terms and over theνterm:

p(ν|ϕ,κ)=Inv-Gamma(ν|ϕ,κ), (7)

$$p(\lambda_{j}|\eta ,\tau )=\text{Inv-Gamma}(\lambda_{j}|\eta ,\tau ),$$ (8)

where Inv-Gamma (*x*|*a*,*b*) denotes an Inverse-Gamma distribution with shapea, scaleb,and modeb​/​(a+1):

$$\begin{array}{ccc} \text{Inv-Gamma}\left(x\text{|}a,b\right)=\frac{b^{a}}{\Gamma \left(a\right)}\frac{1}{x^{a+1}}\text{exp}\left(\frac{-b}{x}\right),a>0,b>0. & & \end{array}$$ (9)

To simplify notation, we will henceforth letλdenote a set of${\left\{\lambda_{j}\right\}}_{j=1}^{J}$parameters and not highlight dependence on terms that remain fixed. We now need to regardλandνas random variables and by doing so the posterior distribution over all variables takes the form:

$$\begin{array}{l} p\left(w,\;\lambda ,\;\nu \;\text{|}\;y,\;X\right)=\frac{p\left(y\text{|}X,w,\nu \right)p\left(w\text{|}\lambda \right)p\left(\lambda \right)p\left(\nu \right)}{∭p\left(y\text{|}X,w,\nu \right)p\left(w\text{|}\lambda \right)p\left(\lambda \right)p\left(\nu \right)\;\;\text{d}w\;\text{d}\lambda \;\text{d}\nu }. \end{array}$$ (10)

This expression cannot be evaluated analytically. In what follows, we will resort to an empirical Bayesian estimation procedure and assume thatp(w|y,X)=∬p(w,ν,λ|X,y)dν dλcan be approximated by$p\left(w\text{|}y,X,\tilde{\nu },\tilde{\lambda }\right),$where$\tilde{\nu }$and$\tilde{\lambda }$are fixed values obtained by maximizing the marginal posterior distribution:

p(λ,ν|  y,X)=∫p(w,λ,ν|  y,X)dw. (11)

We will return to assumptions entailed by this procedure later. Our goal is to use an expectation-maximization (EM) framework (Dempster et al., 1977;Minka, 1998;Neal & Hinton, 1998) to maximize this marginal distribution. In brief, this involves lower boundingln p(λ,ν​|y,X)with a functionℱ(λ,ν,q)that is a function of a valid probability distributionq(w)overw, and a function of the parametersλandν(Bishop, 2006):

$$\begin{array}{ccc} ℱ\left(\lambda ,\nu ,q\right)=\int q\left(w\right)\text{ln}\frac{p\left(w,\lambda ,\nu \text{|}\;y,X\right)}{q\left(w\right)}\text{ d}w. & & \end{array}$$ (12)

The framework involves alternating between an “E-step” (finding a good lower bound) and an “M-step” (subsequently maximizing this bound) (Minka, 1998). Suppose we initialize the algorithm with$\lambda^{\left(k\right)}$and$\nu^{\left(k\right)}$wherek=0. In the expectation step (E-step), we fixλandνto these values and maximize$ℱ\left(\lambda^{\left(k\right)},\nu^{\left(k\right)},q\right)$with respect toq(w). It can be shown that the lower boundℱwill touch the objective when$q\left(w\right)=p\left(w\text{|}\;y,X,\lambda^{\left(k\right)},\nu^{\left(k\right)}\right)\equiv q^{\left(k\right)},$where the posterior distribution ofwgiven a fixed set of parametersλandνtakes the form:

$$p\left(w\text{|}y,X,\lambda ,\nu \right)=\frac{p\left(y\text{|}X,w,\nu \right)p\left(w\text{|}\lambda \right)}{p\left(y\text{|}X,\nu ,\lambda \right)}$$ (13)

                                   =N(w|μ,Σ),

$$\;\Sigma ={\left(\Lambda^{-1}+\nu^{-1}X^{⊤}X\right)}^{-1},$$ (14)

$$\;\mu =\nu^{-1}\Sigma X^{⊤}y.$$ (15)

We will henceforth let$\tilde{\mu }$and$\tilde{\Sigma }$denote mean and covariance of$p(w|y,X,\lambda^{\left(k\right)},\nu^{\left(k\right)})$. Definingq(w)given$\lambda^{\left(k\right)}$and$\nu^{\left(k\right)}$is the E-step. In the maximization step (M-step), we keep this distribution fixed and now maximize$ℱ\left(\lambda ,\nu ,q^{\left(k\right)}\right)$with respect toλandν. Ignoring irrelevant terms, one can show that we need to find a set of parameters$\lambda^{\left(k+1\right)}$and$\nu^{\left(k+1\right)}$that maximizes$E_{q^{\left(k\right)}}\left[\text{ln }\left(p\left(y\text{|}X,w,\nu \right)p\left(w\text{|}\lambda \right)p\left(\lambda \right)p\left(\nu \right)\right)\right]$where$E_{q^{\left(k\right)}}\left[\cdot \right]$denotes expectation under$q^{\left(k\right)}$and where

ln p(y,w,λ,ν|X)=−12ν(y−Xw)⊤(y−Xw)                                                                                                  −M2Inν−12wTΛ−1w−12In|Λ|+∑j=1JIn1λj1+η                                                                                −∑j=1Jτλj+In1ν1+ϕ−κν+constant. (16)

Recall thatwis a column vector that contains all$\beta_{j}$weights and thatΛis a block-diagonal matrix. We know that$p(w|y,X,\lambda^{\left(k\right)},\nu^{\left(k\right)})$is Gaussian with mean$\tilde{\mu }$and covariance$\tilde{\Sigma }$. Let${\tilde{\mu }}_{j}$denote blocks in$\tilde{\mu }$associated with predictor groupj. Further, let${\tilde{\Sigma }}_{j}$denote a block of size$D_{j}\times D_{j}$along the diagonal in$\tilde{\Sigma }$associated with predictor groupj. We again discard irrelevant terms, and now see that the M-step amounts to maximizing the following expression with respect toνandλ:

ℒ=−12ν((y−Xμ˜)⊤(y−Xμ˜)+Tr(X⊤XΣ˜))−M2ln ν               −∑j=1J12λj(μ˜jTΩj−1μ˜j+Tr (Ωj−1Σ˜j))−12∑j=1JDjInλj+∑j=1JIn1λj1+η∑j=1Jτλj+In1ν1+φ−κν. (17)

Finding a set ofλandνparameters that fulfill$\partial ℒ/\partial \lambda_{j}=0$and∂ℒ/∂ν=0yields the following closed-form update rules:

$$\lambda_{j}^{\left(k+1\right)}=\frac{{\tilde{\mu }}_{j}^{⊤}\Omega_{j}^{-1}{\tilde{\mu }}_{j}+\text{Tr}\left(\Omega_{j}^{-1}{\tilde{\Sigma }}_{j}\right)+2\tau }{D_{j}+2\eta +2},$$ (18)

$$\nu^{\left(k+1\right)}=\frac{{\left(y-X\tilde{\mu }\right)}^{⊤}\left(y-X\tilde{\mu }\right)+\text{Tr}\left(X^{⊤}X\tilde{\Sigma }\right)+2\kappa }{M+2+2\phi }.$$ (19)

The procedure of fitting the model thus involves (I) specifying hyperprior parametersϕ,κ,ηandτ, (II) starting with an initial guess for$\lambda_{1},\lambda_{2},...\lambda_{J}$andν, (III) estimating$\tilde{\Sigma }$and$\tilde{\mu }$, (IV) updating$\lambda_{j}$andνaccording toEqs. 18and19, and subsequently iterating between step (III) and (IV) until convergence.

#### 2.1.1 Predictions

The above procedure yields a set of hyperparameters$\tilde{\nu }$and$\tilde{\lambda }$from the training data that may be sensible in several circumstances, and we will consequently focus on$p(w|y,X,\tilde{\nu },\tilde{\lambda })$. For predictive purposes, we assume that the following approximation holds:

$$\begin{array}{l} p(\hat{y}|y,X,\hat{X})\;\approx p(\hat{y}|y,X,\hat{X},\tilde{\nu },\tilde{\lambda })\\ \;\;\;\;\;\;\;\;\;\;\;\;\;\;\;\;\;\;\;\;\;\;\;\;\;\;\;\;\;\;=\int p\left(\hat{y}\text{|}w,\hat{X},\tilde{\nu }\right)p\left(w\text{|}\;y,X,\tilde{\nu },\tilde{\lambda }\right)\text{d}w. \end{array}$$ (20)

That is, to make predictions about a novel response variable,$\hat{y}$, given a new set of predictors$\hat{X}$and inferred model parameters, we focus on$p(\hat{y}|y,X,\hat{X})$and letp(λ,ν|X,y)be represented by a delta function$\delta \left(\nu -\tilde{\nu },\lambda -\tilde{\lambda }\right)$(Tipping, 2001). The resulting distribution is Gaussian with mean$\hat{X}\tilde{\mu }$and covariance$\tilde{\nu }I+\hat{X}\tilde{\Sigma }{\hat{X}}^{⊤}$. The terms$\tilde{\mu }$and$\tilde{\Sigma }$here refer to the estimates inEqs. 14and15, respectively. To make point predictions, we may now focus on the estimated model weights$w=\tilde{\mu }$and simply compute$\hat{X}w$. Our focus will be on such point predictions.

For the interested reader and illustrative purposes, we visualize variability in the estimated weights inFigures 1-3based onEq. 13. It is important to stress that$p(w|y,X,\tilde{\lambda },\tilde{\nu })$is conditional on the point estimates of$\tilde{\lambda }$and$\tilde{\nu }$and that it may fail to be even close to a reasonable approximation of∫∫p(w,ν,λ|X,y)dνdλ(Wolpert & Strauss, 1996).

## 3 Results

### 3.1 Simulations

In addition to evaluating the predictive accuracy of encoding models or decoding models on held-out data, neuroimaging studies often inspect and interpret point estimates of model parameters. In linearized encoding models, model weights are often assumed to be directly interpretable in terms of the underlying brain activity generating a measured response (Haufe et al., 2014). Weights of general linear models (GLMs) in fMRI analysis (Friston et al., 1994) or temporal response functions in M/EEG analysis (Ding & Simon, 2012;Lalor et al., 2009) are prominent examples. However, the choice of regularization can significantly impact the relative magnitude of the estimated weights, in particular in problems with many, potentially correlated, sets of predictors. It can therefore be relevant to simulate data with known target model weights in order to better understand the properties of regularized regression estimators and identify situations where they can be useful or can complicate interpretation.

Below, we consider different simulations to illustrate the properties of the described model. We compare the proposed EM-based banded regression estimator (henceforth EM-banded) with a standard Ridge regression estimator:$w={\left(X^{T}X+\alpha I\right)}^{-1}X^{T}y$(Hoerl & Kennard, 1970). To simplify readability, we consider simulations where we fix the parameters related to the Inverse-Gamma priors to the same value,γ, such thatτ=η=κ=ϕ=γ. Notice that the hyperpriors become broad whenγapproaches zero.

#### 3.1.1 Simulation 1: Suppressing contributions from a group of predictors

In models with groups of predictors representing different stimulus feature spaces, it is sometimes needed to identify entire feature spaces that can be excluded from an analysis without compromising predictive accuracy. To simulate such a situation, we here define three stimulus feature spaces,$F_{1}$,$F_{2}$, and$F_{3}$, and one response variableywhich contains mixed versions of$F_{1}$and$F_{3}$, but not$F_{2}$($F_{2}$does not contribute to the response). We thus simulate the response as$y=F_{1}w_{1}+F_{3}w_{3}+\epsilon$, whereϵis a Gaussian noise term. In this situation, we thus need the estimator to identify (close to) zero weights for$F_{2}$. Each feature space has 64 predictors and a total ofM=1024observations are simulated. The weights,$w_{1}$and$w_{3}$, as well as the feature space$F_{1}$and$F_{3}$, are drawn from Gaussian distributions. The mixed target signal and the noise term have equal power. We compare weights estimated by the EM-banded model with weights estimated by Ridge regression models. To exemplify how hyperparameters controlling Inverse-Gamma hyperpriors can impact the estimated weights, we fit models forγfixed to$10^{-4}$,$10^{-3}$,$10^{-2}$, and$10^{-1}$. The Ridgeαhyperparameter is set to$10^{4}$,$10^{3}$,$10^{2}$, and$10^{1}$. The terms$\Omega_{1}$,$\Omega_{2}$, and$\Omega_{3}$are identity matrices.

**Fig. 1.**
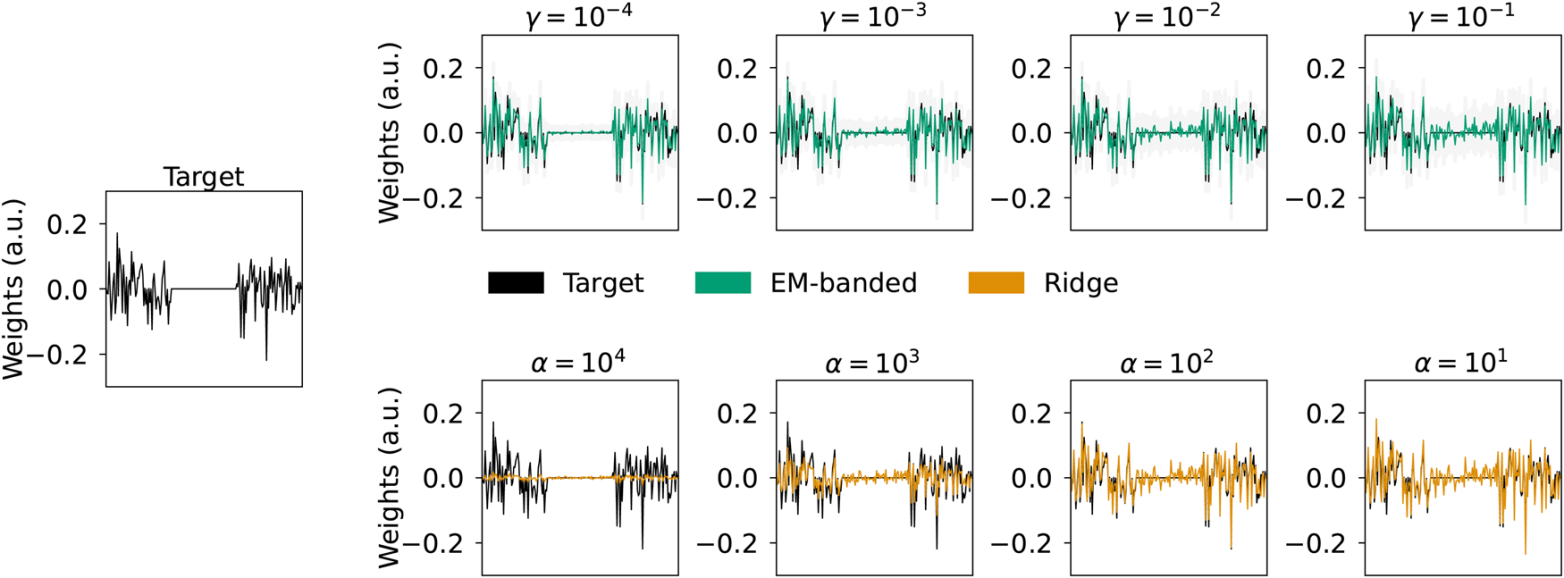
Simulation 1 illustrating regularization properties in a regression problem with three groups of predictors. Left middle panel: simulated target weights (black). The predictors are divided into three distinct predictor groups,$F_{1}$,$F_{2}$, and$F_{3}$. Target weights associated with$F_{2}$are equal to zero. Top row: weights estimated with EM-banded model for different values ofη=ϕ=τ=κ=γ. Shaded gray errorbars depict 1%, respectively, 99% percentile of samples from a multivariate Gaussian with mean and covariance defined according toEq. 13. Bottom: weights estimated with Ridge regression model for different Ridge regularization parametersα. The target weights are shown in black.

Figure 1shows results from Simulation 1. The Ridge regression model yields “dense” solutions for all considered Ridge hyperparameters,α, with no dramatic overall scale differences between weights associated with$F_{1}$,$F_{2}$, or$F_{3}$. In contrast, the EM-banded model excessively shrinks weights associated with feature set$F_{2}$for smaller values ofγ. As expected, the EM-banded model tends to show signs of under-shrinkage when the Inverse-Gamma prior distributions have little mass near zero and in these cases, the overall scale differences between the different sets of weights associated with$F_{1}$,$F_{2}$, and$F_{3}$are smaller.

#### 3.1.2 Simulation 2: Encouraging smoothness for a feature subset

It is sometimes relevant to encourage smoothness on sets of weights, for instance, in analyses that approximate smooth FIR filters. An example hereof is fMRI encoding analyses that model the relation between a task-regressor augmented with multiple time lags and the BOLD voxel response. In this case, it can*a priori*be expected that the approximated FIR filter should be smooth due to hemodynamic properties of the BOLD signal (Goutte et al., 2000). It can similarly be relevant to assume smoothness across spectral channels in analyses that model relations between average stimulus power spectra and BOLD fMRI responses.

Here, we explore one way of encouraging smoothness using the definition of$\Omega_{j}$inEq. 6. To illustrate the effect of$\Omega_{j}$, we consider a simulated regression problem with two feature spaces,$F_{1}$and$F_{2}$. Each feature space has 64 predictors and is drawn from a Gaussian distribution. Data are simulated as$y=F_{1}w_{1}+F_{2}w_{2}+\epsilon$, whereϵis drawn from a Gaussian. A total ofM=1024observations are simulated. The weights associated with the first feature space$w_{1}$are simulated to be sinusoidal (i.e., smooth), while$w_{2}$weights are Gaussian. The signal-to-noise ratio (SNR) is -5 decibels (dB) and we fit the EM-banded models withγfixed to a low value of$10^{-4}$(i.e., corresponding to a broad hyperprior). The$\Omega_{1}$term associated with$F_{1}$is parameterized according toEq. 6with$h_{1}$set to1,5, and10to illustrate its effect.

**Fig. 2.**
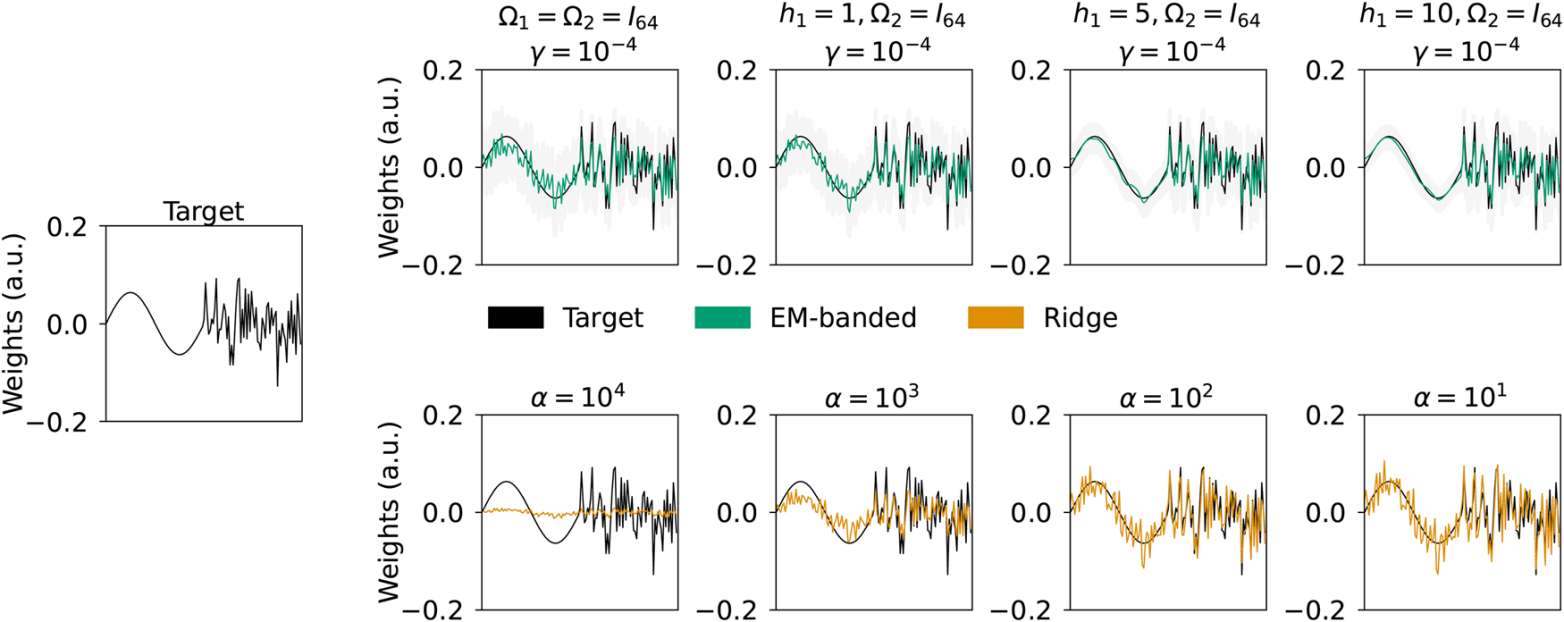
Simulation 2 illustrating effects of smoothness encouragement. Left middle panel: simulated target weights (black) divided into two distinct predictor groups,$F_{1}$and$F_{2}$. Weights associated with$F_{1}$are sinusoidal, while weights associated with$F_{2}$are drawn from a Gaussian. Top row: weights estimated with the EM-banded model for$\gamma =10^{-4}$but with different parameterizations for$\Omega_{1}$(defined inEq. 6). Specifically, we change the$h_{1}$term in this equation in order to encourage smoothness. Shaded gray errorbars depict 1%, respectively, 99% percentile of samples from a multivariate Gaussian with mean and covariance defined according toEq. 13. Bottom: weights estimated with Ridge regression model for different Ridge regularization parametersα. The target weights are shown in black.

Figure 2shows weights estimated with Ridge estimators for differentαvalues (below) and weights estimated by the EM-banded estimators (above). At higher levels of$h_{1}$, the EM-banded estimator approximates the weight distribution of the first feature space$F_{1}$with high accuracy. The Ridge estimator, on the other hand, yields weights that tend to show more rapid (high frequency) fluctuations across neighboring weights associated with$F_{1}$compared to the target weights.

#### 3.1.3 Simulation 3: Encouraging differential shrinkage of individual weights

In Simulation 1, the EM-banded model was used to shrink weights of an entire feature space (i.e., groups of predictors). The model can similarly encourage differential shrinkage of individual weights. To illustrate this, we consider a simulation where we assume that the number of groups equals the number of predictors (i.e., each group contains a single predictor). We simulate 512 predictors,$F_{1}$,$F_{2}$,…,$F_{512}$, and a response variableywhich contains mixed versions of some of these predictors. A total ofM=1024observations are simulated. It is assumed that$y=\sum_{j}F_{j}w_{j}+\epsilon$whereϵis Gaussian noise. We assume that a high proportion of the target weights$w_{j}$is equal to zero. Each row in$\left[F_{1},F_{2},...,F_{512}\right]$is drawn from a multivariate Gaussian with zero mean and a covariance defined as$C=\text{exp}\left(-0.3\text{|}\;k-i{|}^{2}\right),k=1,...,512,i=1,...,512$. The SNR is equal to 0 dB. We again compare Ridge regression model fits with EM-banded model fits using the same set of fixed hyperparameters as in Simulation 1.

The estimated weights for the Ridge and EM-banded model are shown inFigure 3. It is evident that the EM-banded estimator with$\gamma =10^{-4}$excessively shrinks several of the individual weights. In effect, the EM-banded model recovers the target weights with relatively high accuracy. In comparison, the Ridge estimator tends to dilute effects across neighboring (correlated) predictors and pull weights toward zero whenαattains high values.

**Fig. 3.**
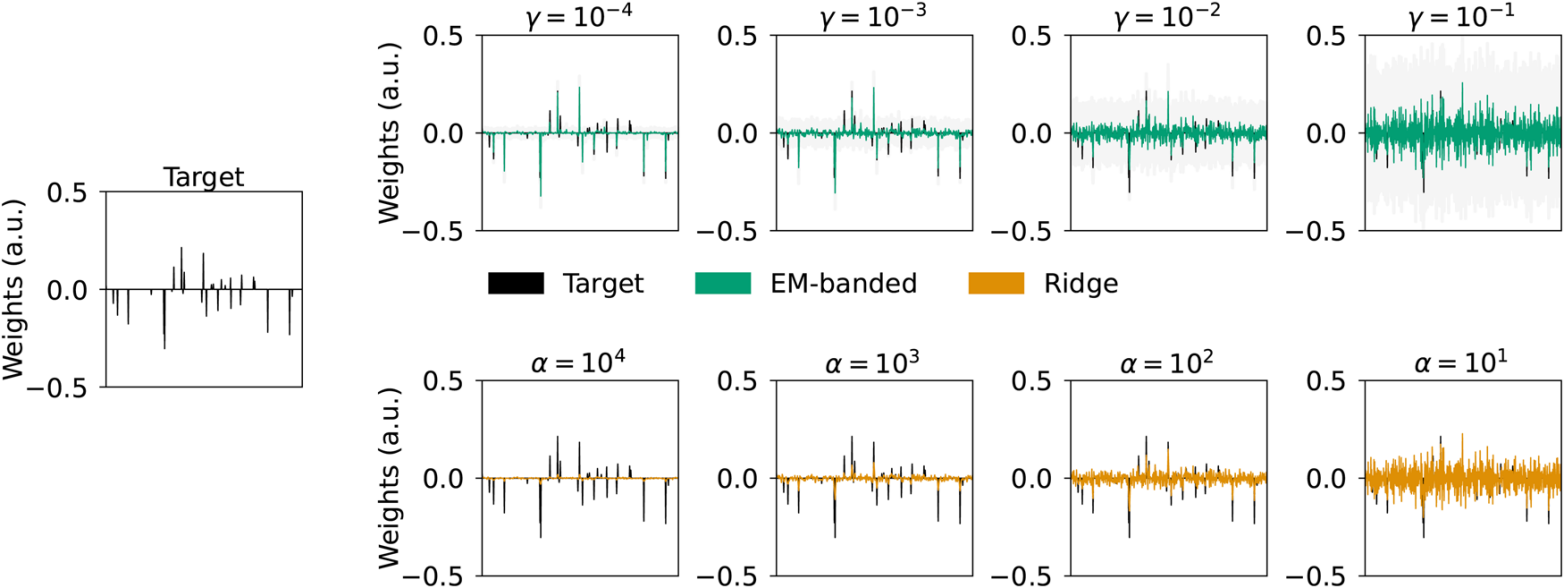
Simulation 3 illustrating differential shrinkage of individual weights. Left middle panel: simulated target weights (black). Top row: weights estimated with EM-banded model for different values ofη=ϕ=τ=κ=γ. Shaded gray errorbars depict 1%, respectively, 99% percentile of samples from a multivariate Gaussian with mean and covariance defined according toEq. 13. Bottom: weights estimated with Ridge regression model for different Ridge regularization parametersα. The target weights are shown in black.

#### 3.1.4 Simulation 4: Interpretation of weights with correlated predictors

Until now, we have not explored how strong correlations among groups of predictors can impact results. Models involving correlated groups of predictors occur often in EEG or fMRI encoding models that model responses to naturalistic stimuli (Hamilton & Huth, 2020). To exemplify this situation, this simulation considers the example of predicting responses to natural speech. Speech signals can be characterized by their lower level acoustic features as well as higher level phonetic or semantic features (Broderick et al., 2019;Huth et al., 2016). The different hierarchical levels can each be modeled by multidimensional features, but the statistics of natural speech imply a degree of correlation between such feature groups (see, e.g., panel B inFig. 4). This can complicate inferences about neural responses unique to a given group.

**Fig. 4.**
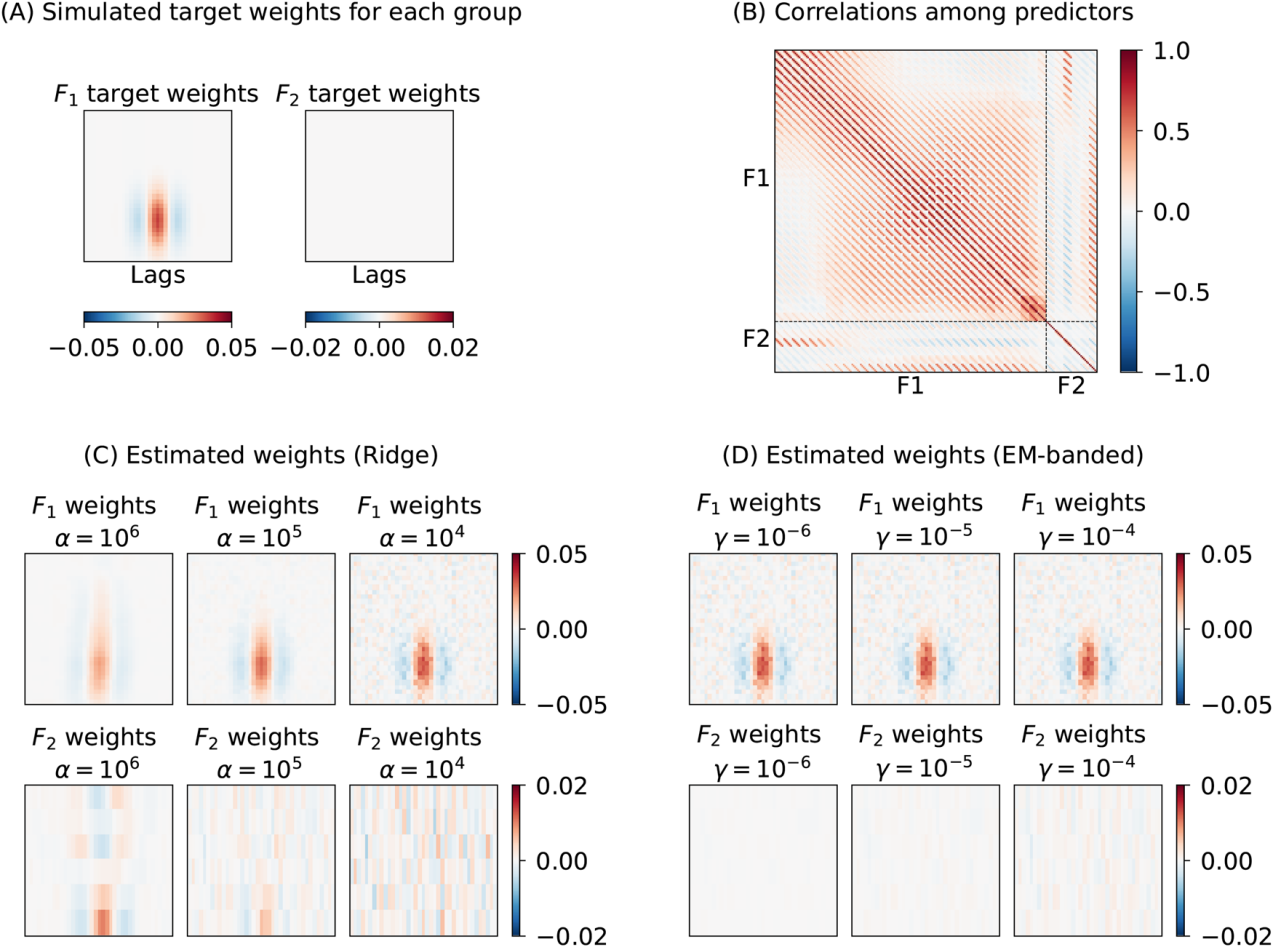
Simulation 4 illustrating behavior of the Ridge estimator and the EM-banded estimators when there are strong correlations among groups of predictors,$F_{1}$and$F_{2}$. The target variable is simulated as$y=F_{1}w_{1}+\epsilon$. (A) Target weights associated with each of the two feature sets. (B) Matrix of Pearson correlation coefficients between predictors. Dashed lines are used to highlight predictor groups,$F_{1}$and$F_{2}$. (C) Weights estimated with Ridge estimator for each of the two groups. (D) Weights estimated with the EM-banded estimator for each of the two groups. Ridge estimators withαset to$10^{6}$,$10^{5}$, and$10^{4}$are shown. EM-banded estimators were considered withγset to$10^{-6}$,$10^{-5}$, and$10^{-4}$.

To illustrate this, we simulate responses to speech acoustic and phonetic features extracted from natural speech audio (Garofolo et al., 1993) (see Section 4 in theSupplementary Materialfor details on feature extraction). A first feature set$F_{1}$contains time-lagged spectrogram features and$F_{2}$contains time-lagged regressors created from phonetic annotations. We simulate a response as$y=F_{1}w_{1}+\epsilon$, that is, only the feature set associated with the spectrogram features$F_{1}$affects the response variable,y. The SNR is equal to 0 dB and the target filter weights,$w_{1}$, are shown inFigure 4A. We again compare Ridge and EM-banded regression model fits using a set of fixed hyperparameters.

Figure 4shows the results of this simulation. The EM-banded estimator accurately recovers the weights associated with the speech spectrogram$F_{1}$while excessively shrinking the weights associated with the phonetic features$F_{2}$, as desired. The Ridge estimator, on the other hand, pulls weights associated with correlated predictors in the two feature groups toward each other at higher levels of regularization. This has the undesired consequence that a temporally located response function associated with the phonetic feature group$F_{2}$emerges. This can complicate the interpretation of the weights associated with$F_{2}$, for example, if such point estimates of weights were interpreted or further analyzed in second-level inferences. Simulating the opposite case, that is, simulating only responses to phonetic features (and no contribution from spectrogram features), leads to the equivalent result: weights associated with the spectrogram feature group show temporally located response functions (see Section 4 in theSupplementary Material). The described effect depends on the degree of correlation among predictors and on the amount of data available for fitting the models. We note that high levels of regularization often occur in practice for Ridge estimators, for instance, when tuning the regularization term to maximize the Pearson correlation coefficient between model predictions and target variables (due to its insensitivity to scaling and shift mismatches). For a related, but more stylized simulation, see Section 5 in theSupplementary Material.

**Fig. 5.**
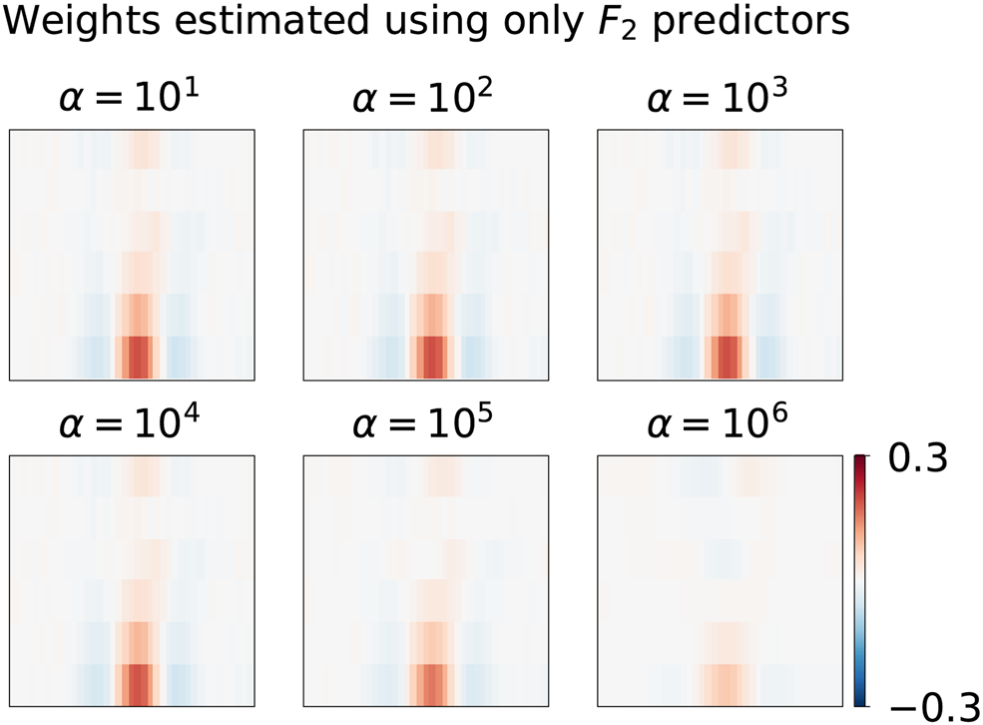
Overlooking important features in a Ridge regularized encoding model. The outcome variable is again simulated as$y=F_{1}w_{1}+\epsilon$, that is, the simulated target is simulated to be affected by$F_{1}$and not$F_{2}$, but only$F_{2}$is included in the prediction model. Panels reflect estimates with different Ridge regularization strengths.

This simulation highlights a potential source of pitfall for the interpretation of model weights in situations with co-varying features affecting the response. To illustrate this further, consider again the simulation where the response is driven by the first feature set$F_{1}$, but now only the correlated set$F_{2}$is included in the model.Figure 5depicts the estimated weights by Ridge models in this case. The model with only$F_{2}$predictors now misleadingly indicates a temporally located response for all of the consideredαparameters, despite the fact that the target regression weights associated with$F_{2}$are zero. Interpretation of the reduced model is clearly different from the model that includes both groups of predictors.

#### 3.1.5 Simulation 5: Visualizing behavior under unfavorable signal-to-noise ratios

As illustrated in Simulation 3, the EM-banded estimator can be used to encourage differential shrinkage of weights associated with individual predictors. The behavior of such an estimator will depend on several factors, including the amount of data available to support the identification ofλ, correlations among predictors, and the SNR. Here, we illustrate the behavior of the EM-banded estimator in a simulation with poor SNR. We simulate two predictors$F_{1}$and$F_{2}$and a response variable$y=F_{1}w_{1}+F_{2}w_{2}+\epsilon$. The predictors as well as the noise termϵis drawn from Gaussian distributions. The target weights are fixed to non-zero values. The SNR is approximately−20dB. We consider simulations where the number of observations is512,1024, and8192, and we fit EM-banded models to the data from each simulation. We focus on estimators with$\gamma =10^{-4}$, respectively.$\gamma =10^{-2}$to illustrate how differences in these prior choices affect the estimated parameters. This procedure is repeated many times to elucidate the effect.

**Fig. 6.**
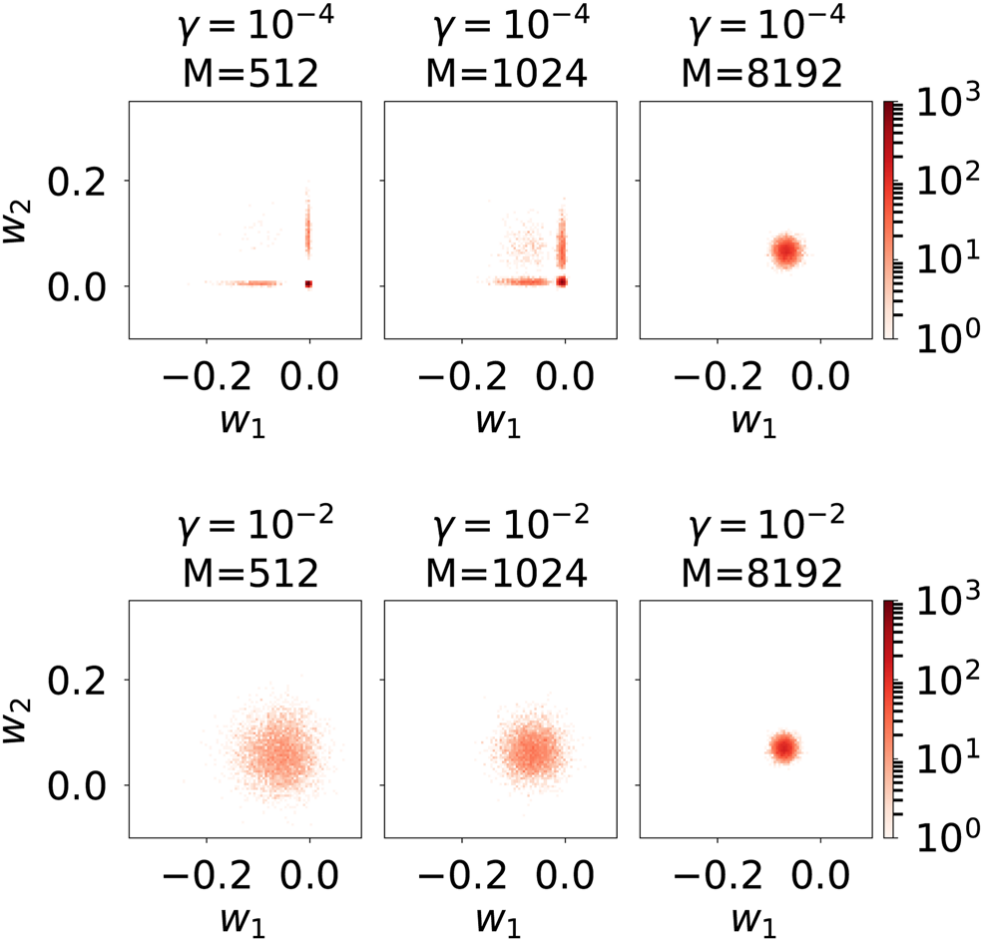
Simulation 5 illustrating regularization properties with only two predictors and a poor SNR. 2D histogram visualizations of estimated regression weights,$w_{1}$and$w_{2}$. Columns show simulations with increasing number of observations. Top row: weights estimated with the EM-banded estimator with$\gamma =10^{-4}$. Bottom row: weights estimated with the EM-banded estimator with$\gamma =10^{-2}$.

Figure 6shows 2D histogram visualizations of the estimated weights$w_{1}$and$w_{2}$from these simulations. Notably, the estimator with the lower$\gamma =10^{-4}$tends to exhibit excessive shrinkage to either of the two weights when the number of observations is low (M=512). Such over-shrinkage is undesirable. The estimator even collapses to$w_{1}\approx w_{2}\approx 0$occasionally, which again is undesirable. Increasingγto$10^{-2}$here yields dramatically different estimates and less differential shrinkage of the two weights (increasingγencourages solutions that allow for less excessive shrinkage). These simulations underline subtleties that can yield dramatic effects on the estimated weights, which is important to keep in mind when focusing on “default” priors for$\lambda_{j}$.

### 3.2 fMRI encoding analysis with “stimulus-irrelevant” predictors

Building encoding models often involves deciding on which of multiple potentially relevant feature sets to include as predictors. Choosing features both implies a risk of overlooking feature sets that are potentially relevant, and including feature sets that are irrelevant for predicting the target variables. In this section, we investigate how the inclusion of stimulus-irrelevant predictors - here defined as simulated noise predictors that are unrelated to the task or stimuli - can impact results in an fMRI encoding analysis and how the EM-banded estimator may help suppress their contribution.

This example uses a publicly available BOLD fMRI dataset that has been described inNakai et al. (2021,^2022^). The dataset contains BOLD fMRI data acquired from five participants listening to excerpts from music in 12 training runs and 6 test runs. Informed consent had been obtained from all participants prior to their participation, and procedures were approved by local ethics and safety committees, seeNakai et al. (2021,^2022^). Each functional run lasted 10 min and consisted of 40 musical clips that each was 15 s long. We focused on encoding models with three feature sets,$F_{1}$,$F_{2}$, and$F_{3}$. The first two feature sets,$F_{1}$and$F_{2}$, were related to the music stimuli: a 10-dimensional genre-label feature given byTzanetakis and Cook (2002)indicating which of 10 musical genres a given stimulus is assigned to ($F_{1}$), and a time-averaged 32-dimensional spectrogram of the audio stimulus ($F_{2}$). As the third feature set ($F_{3}$) unrelated to the stimuli, we included a 400-dimensional multivariate Gaussian noise.$F_{1}$,$F_{2}$, and$F_{3}$were considered separate groups for the EM-banded model. For simplicity, we always initialize the EM algorithm with$\lambda_{j}$andνparameters set to1and define that no more than200iterations should be considered. It may sometimes be advisable to use different initialization strategies. More details related to fMRI preprocessing, audio feature extraction, and model fitting are described in Section 1 in theSupplementary Material.

**Fig. 7.**
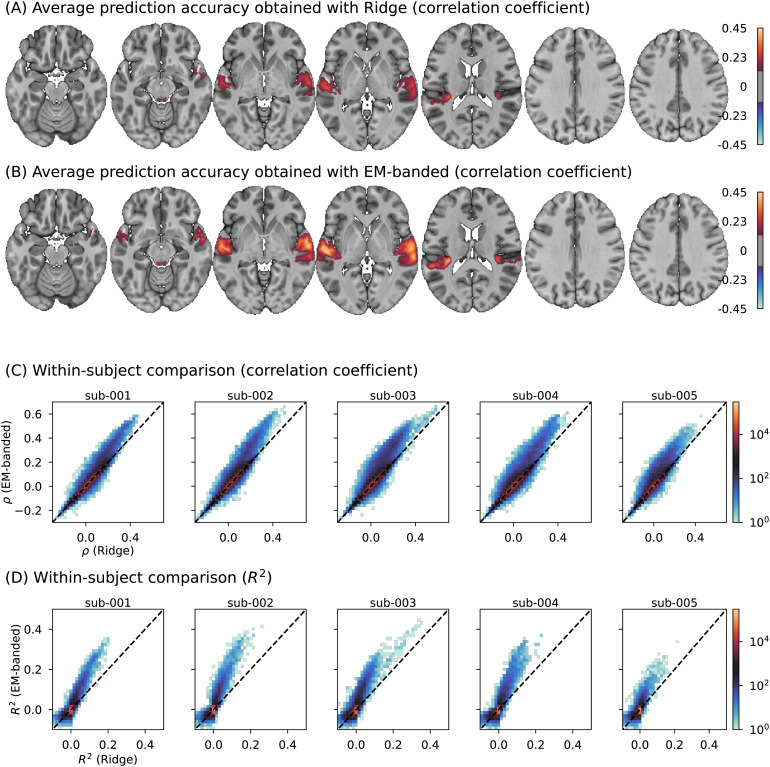
A BOLD fMRI encoding analysis where stimulus-irrelevant predictors are included in the analysis. (A) Group-mean prediction accuracies for models trained with the EM-banded framework. (B) Group-mean prediction accuracies for cross-validated Ridge regression models. Maps in (A) and (B) have been arbitrarily thresholded for visualization purposes. (C) 2D histogram visualization of prediction accuracies (Pearson correlation coefficient,ρ) for the EM-banded and Ridge estimators for each subject. The dashed line indicates similar performance across models. (D) Similar visualizations as in (C) but using$R^{2}$as performance metric.

Figure 7shows the predictive accuracies obtained with the Ridge estimator and the EM-banded estimator. Predictive accuracy is here evaluated both as Pearson correlation coefficient between the predicted and measured BOLD fMRI response data, and as explained variance ratio. Compared to the Ridge estimator, the EM-banded estimator yielded higher predictive accuracy for all subjects in both metrics (allp<0.05, Wilcoxon signed-rank tests across all voxels used for fitting models). The improvement in terms of Pearson correlation coefficient among the top 10000 voxels was (mean±standard deviation):0.0335±0.0515(sub-001),0.0411±0.0629(sub-002),0.0568±0.0699(sub-003),0.0391±0.0637(sub-004), and0.0298±0.0522(sub-005).

The regularization termαof the Ridge estimator varies substantially across voxels. This can occur, for instance, if voxels with higher signal-to-noise ratio require less overall regularization. Changes inαimpacts the overall scale of all weights. This can lead to misleading spatial weight maps indicating effects of predictors that are stimulus irrelevant. This is illustrated inFigure 8showing the group-mean standard deviation of predictions obtained with stimulus-irrelevant predictors. The predictions were obtained using only the task-irrelevant predictors to predict voxel responses (discarding the spectrogram and genre features) with the Ridge estimator. As can be seen, the standard deviation of these predictions is high specifically in auditory cortical regions. This may reflect that the Ridge estimator declares less overall shrinkage in voxels in regions with higher SNR. In effect, the map can misleadingly be interpreted to suggest regional auditory-evoked activation to a random feature*because of*the regularization. Encouraging differential shrinkage of each feature space with the EM-banded estimator avoids this problem, showing no clear spatial patterns in these maps.

**Fig. 8.**
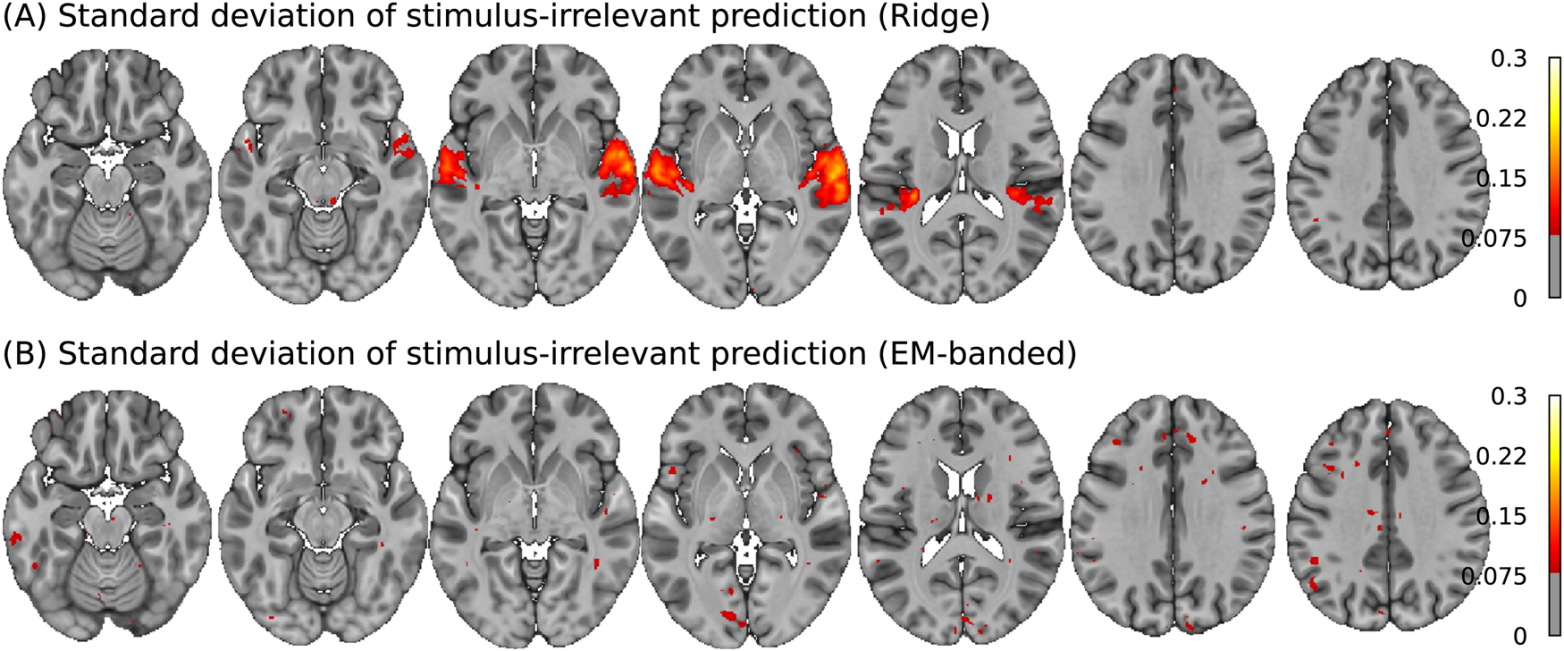
Group-mean standard deviations of predictions with a stimulus-unrelated noise feature estimated with Ridge regression (A) and the EM-banded framework (B).

### 3.3 EEG-based decoding analysis

In this example, we consider a decoding analysis of single-trial EEG responses to continuous natural speech. The example follows a popular approach of predicting the continuous speech amplitude envelope using linear combinations of time-lagged EEG scalp electrode responses at lower frequencies (Broderick et al., 2018;Crosse et al., 2016;Ding & Simon, 2012;O’Sullivan et al., 2014;Wong et al., 2018). In this approach, the goal is to find a multidimensional filterwthat linearly maps from (features in) the multichannel EEG response to the envelope of the speech stimulusy. The decoding analysis can be formulated as$y\left(t\right)=\sum_{c}\sum_{t}x\left(t-\tau ,c\right)w\left(c,\tau \right)+\epsilon$, wherex(t−τ,c)denotes preprocessed electrode response in channelcat time pointt−τand whereτindicates the time delay between stimulus and response. The model can be specified by concatenating multiple time-lagged versions of each EEG electrode response in a design matrixXinEq. 1. The regression problem now amounts to estimating coefficients of a multi-dimensional FIR filter (de Cheveigné et al., 2018). In this context, it is natural to consider each time-lagged electrode response as a feature group$F_{j}$inEq. 2and allow for differential shrinkage of weights associated with each group. For instance, it can be relevant to allow for excessive shrinkage of a noisy or seemingly irrelevant electrode for all its time-lag copies.

While EEG speech decoding studies often focus on low-frequency activity (<15Hz), it is known from intracranial recordings that the power of higher frequency neural activity in the gamma range also tracks features of speech stimuli (Crone et al., 2001;Mesgarani & Chang, 2012;Pasley et al., 2012). These frequencies are typically highly attenuated in scalp EEG. Nonetheless, including high gamma power features has been shown to improve predictive accuracy of EEG decoding models for some subjects (Synigal et al., 2020). However, including more EEG features, some of which may have very low SNR, is also be associated with a risk of adding predictors to the decoding model that introduce noise to the predictions. It may thus be desirable to allow for differential shrinkage of different EEG features. In this example, we therefore explore predictive accuracy of the EM-banded model when both lower and higher frequency EEG features are included in such a decoding analysis.

This example uses publicly available EEG data (https://doi.org/10.5061/dryad.070jc) described inBroderick et al. (2018)andDi Liberto et al. (2015). In brief, 19 subjects listened to excerpts from an audiobook while continuous EEG data from 128 scalp electrodes were recorded. All procedures were undertaken in accordance with the Declaration of Helsinki and were approved by local ethics committees, seeBroderick et al. (2019)andDi Liberto et al. (2015). Data were acquired in 20 trials each approximately 180 s in length. We fit decoding models again using the EM-banded framework compared to standard Ridge regression. For both models, we used time-lagged low-frequency (LF) EEG features (1-8 Hz) as well as time-lagged high-frequency (HF) EEG power features (25-35 Hz) (Giraud & Poeppel, 2012) to predict a speech envelope feature. Further details related to EEG preprocessing, audio feature extraction, and model fitting are described in Section 2 in theSupplementary Material. Note that cross-validation was only used for hyperparameter tuning in the Ridge model, but that both models always were tested on held-out data.

**Fig. 9.**
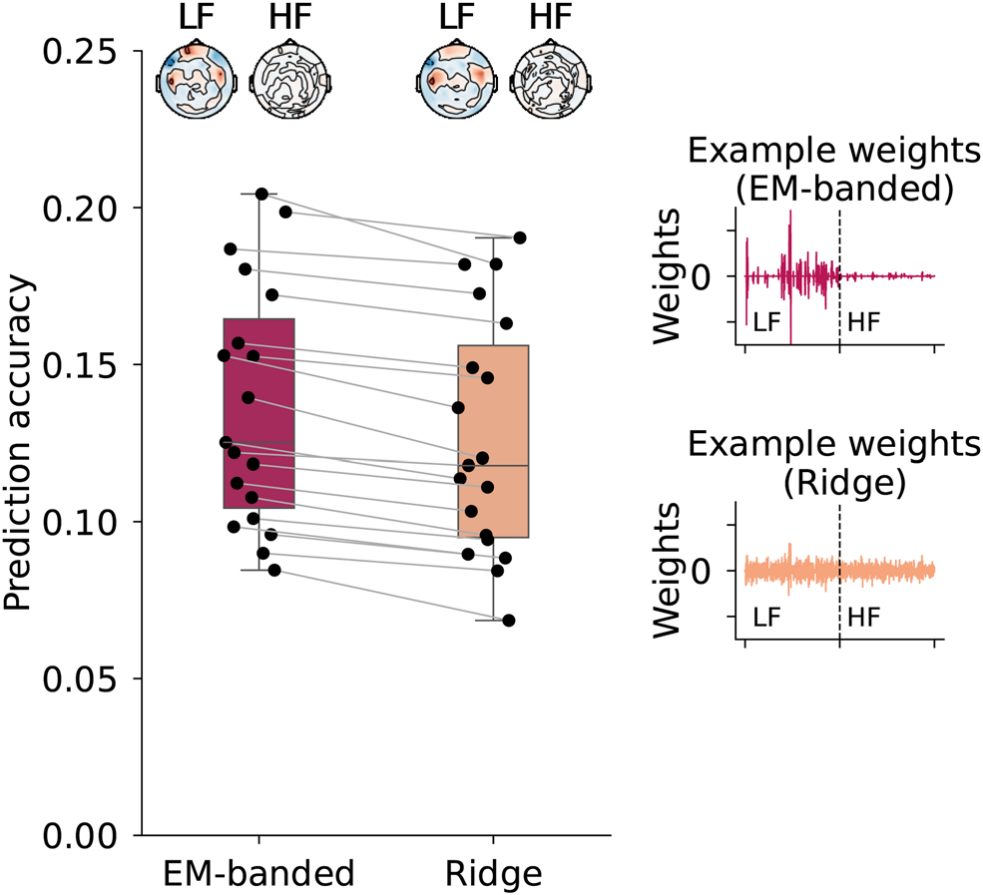
Results from the EEG decoding analysis. Left: boxplot of prediction accuracies obtained with each model. Each point reflects data from a given subject. Prediction accuracy was defined as the average correlation coefficient between the target envelope and decoded envelope in held-out trials. Topographies depict weights of each model at a single time lag for illustrative purposes. The weights are here first normalized by their standard deviation and averaged across participants. Topographies are shown both for weights associated with low-frequency (LF) features and high-frequency (HF) features. Right: Examples of regression weights for one participant.

The results from the analysis are shown inFigure 9. The prediction accuracies obtained with the EM-banded estimator are modestly but consistently higher than the Ridge estimates (p<0.01, Wilcoxon signed rank test). The average correlation between the predicted speech envelope and the target envelope is0.1368±0.0383for the EM-banded model and0.1267±0.0.0377for the Ridge model. The EM-banded approach excessively shrinks groups of weights, whereas the Ridge estimator yields a denser distribution of weights.Figure 9(right) shows an example of weights estimated by the two models for one subject. This illustrates how the overall scale of weights associated with the HF features and LF features is comparable for the Ridge estimator whereas the EM-banded estimator differentially shrinks both weights associated with HF features and groups of LF features associated with single electrodes. Given that high-frequency EEG features are weak and in some subjects not even measurable by scalp electrodes, including HF features can negatively model impact predictions, in particular with a Ridge estimator. Differential shrinkage of groups of weights may in such cases allow for better exploitation of potentially informative HF features.

## 4 Discussion

We explored an empirical Bayes framework for estimating “banded”-type encoding and decoding models. The framework can allow differential shrinkage of groups of weights associated with different groups of predictors (e.g., feature “bands”). The model can further be used to encourage smoothness on such groups of weights. In the following, we discuss modeling considerations and model limitations.

### 4.1 Encoding and decoding models with correlated predictors

Regression models with highly correlated predictors frequently occur in encoding and decoding analyses. Features of continuous naturalistic stimuli (e.g., video or speech) used as predictors in encoding models are often highly correlated. Continuous scalp EEG channels used as predictors in decoding models are typically also highly correlated, and pairs of channels can commonly exceedρ=0.8with high-density EEG recordings.

The described estimator may encourage solutions where subsets of correlated predictors are excessively shrunken. However, the true posterior of weights in analyses with many correlated predictors may show complex multimodal distributions, and point estimates of model parameters (or, approximate conditional distributions) should be interpreted with caution (see, e.g., Simulation 5). Spurious excessive shrinkage of a given feature set in an encoding analysis with correlated feature sets could, for example, be misinterpreted as indicating that the feature is not encoded in a response where it is contributing weakly. Such issues can be particularly difficult to identify when there are many outcome variables (e.g., different voxels with different noise profiles), and little training data to support the identification of model parameters.

Shrinkage procedures can complicate interpretation of weights in encoding analyses with correlated predictors, as illustrated in our simulations. In decoding models, model prediction accuracy on held-out data is often a primary measure. If the main goal is to optimize model performance for some application (e.g., a decoding model optimized for BCI performance), then the choice of shrinkage procedure may be less critical for interpretation as long as it does not compromise performance or lead to exclusion of important features. However, another goal may be to correlate prediction accuracies with behavior or clinical outcome measures. Here, the choice of shrinkage procedure can affect group-level inferences. For instance, multi-channel EEG data may show distinct noise characteristics in different individuals (e.g., noisy electrodes or attenuation of high-frequency activity due to head anatomy). A given shrinkage procedure might differentially impact decoding accuracies across individuals, potentially complicating the interpretation of group-level correlations with behavior.

With highly correlated predictors, it can sometimes be relevant to transform (groups of) the predictors using data dimensionality reduction techniques (de Cheveigné, 2021;de Cheveigné & Parra, 2014;de Cheveigné & Simon, 2008;de Cheveigné et al., 2019;Hyvärinen & Oja, 2000;Zou et al., 2006) and then fit models using the transformed data. This can potentially reduce computational burden without compromising predictive accuracy. Dimensionality reduction can be combined with banded regularization procedures to allow differential shrinkage of regression weights associated with each component. The described framework can also be used in the context of feature selection (Bair et al., 2006;Fan & Lv, 2008;Guyon & Elisseeff, 2006;Neal & Zhang, 2006), for instance, to inform about which stimulus-response lags to consider. Whether the assumptions entailed by such procedures are reasonable again depends on the specific research goals.

Several of our simulations assumed that there is a set of “true” regression weights as defined by data-generating equations. For example, in Simulation 4 we assumed that only one of two correlated feature groups drives a response. Such data-generating processes are deliberately oversimplistic in order to illustrate properties of the model, but are unlikely to reflect realistic scenarios. In naturalistic scenarios, correlated data are likely to be generated by a chain of latent causes. For example, in encoding analyses with different sets of highly correlated features (e.g., different high- and low-level features derived from natural speech), it is conceivable that the different stimulus features as well as the target regression variables each is generated by one of more underlying variables unknown to the modeler. Therefore, regression weights do not allow straightforward causal interpretation in such scenarios, and banded regression procedures do not solve such issues.

### 4.2 Computational burden

Encoding and decoding analyses often involve high-dimensional regression problems. In encoding models, the stimulus or task feature selection is often motivated by some pre-defined hypotheses. Yet, even for well-defined hypotheses, feature sets often translate to a high number of predictors. This, in turn, implies a high computational burden. The proposed EM-banded model involves matrix inversions (often with complexity$O\left(D^{3}\right)$unless specific structure of the matrix can be exploited) which may limit the relevance if the number of predictors is very high. This problem is further amplified in situations with many response variables. Algorithms described inla Tour et al. (2022);Nunez-Elizalde et al. (2019)are well optimized for fMRI encoding analyses with many predictors and a large number of response variables (voxels). For this situation, Section 3 in theSupplementary Materialpresents an alternative formulation of the EM-banded model where it is assumed that the$\lambda_{j}$andνparameters are shared across multiple outcome variables. This formulation allows for a more efficient implementation in scenarios with many outcome variables. Runtime test examples can be found athttps://github.com/safugl/embanded.

### 4.3 Risks of under-shrinkage and over-shrinkage

The described model applies independent Inverse-Gamma priors on$\lambda_{j}$terms associated with different groups of predictors. The hyperpriors could also be omitted and replaced by a constant which will lead to typical type-II maximum likelihood estimates. Several efficient algorithms have specifically addressed such scenarios (Cai et al., 2021;Tipping & Faul, 2003;Wipf & Nagarajan, 2007). The choice of hyperpriors, or lack hereof, can have a large impact on the results and misspecified hyperpriors can be associated with over-shrinkage or under-shrinkage, as exemplified in our simulations. In both cases, this may compromise both predictive accuracy and interpretability of model weights.

In the encoding and decoding analyses examples, we focused on Inverse-Gamma hyperpriors with hyperparameters fixed to$\tau =\eta =\kappa =\phi =10^{-4}$, which corresponds to broad hyperpriors. Choosing broad hyperpriors is convenient in several practical analysis situations, allowing for excessive shrinkage of (groups of) predictors while also putting prior mass on larger values for$\lambda_{j}$andν. Moreover, fixing hyperparameters allows for determination of$\lambda_{j}$andνterms from training data alone without having to tune hyperparameters using cross-validation. This can be particularly convenient in banded regression problems with many feature groups. Section 7 in theSupplementary Materialshows out-of-sample predictive accuracies for EM-banded models with various different hyperpriors fit to data from three of our simulations. Here, the predictive accuracies tend to plateau asγapproaches zero, suggesting that broad hyperpriors are reasonable choices in these simulations.

In many practical applications, a broad hyperprior can be chosen to provide an initial “default” reference model that can be used as a starting point for subsequent model comparisons (Gelman, 2006). In the context of encoding and decoding analyses (where focus mostly is on out-of-sample predictive accuracy), such reference models can be highly useful for identifying cases where certain regularization properties are inappropriate. This procedure could be taken, for example, when exploring whether grouping structures or smoothness constraints are relevant. The EM-banded model offers one estimator among several others that can be considered in such a process.

### 4.4 Empirical Bayes and related work

We explored a parametric empirical Bayes framework (Efron & Morris, 1973;^1975^;Robbins, 1964;MacKay, 1992,^1996^;Morris, 1983) in the context of encoding and decoding analyses. We used this framework to tune regularization hyperparameters from the data and subsequently focus on moments in a conditional distribution, an idea that is widely adopted in hierarchical analyses of neuroimaging data as outlined byFriston, Penny, et al. (2002). Parametric empirical Bayes has been considered in the context of MRI decoding analyses (Sabuncu & Van Leemput, 2011;Wen et al., 2019), in M/EEG source localization problems (Cai et al., 2018,^2021^;Friston et al., 2008;Owen et al., 2008;Owen et al., 2012;Wipf et al., 2010), in multi-subject hierarchical fMRI analyses (Friston, et al., 2002), in MRI segmentation (Iglesias et al., 2015;Puonti et al., 2016,^2020^), and in receptive field models of cell recordings (Park & Pillow, 2011). Likewise, dividing variables into groups and encouraging group-wise sparsity via the group-lasso (Yuan & Lin, 2006) have also been used in M/EEG source localization problems (Haufe et al., 2008;Lim et al., 2017;Ou et al., 2009) and in causal discovery (Bolstad et al., 2011;Haufe et al., 2010;Tank et al., 2017). The group-lasso is convenient in the latter situations because it promotes truly (group-wise) sparse solutions unlike the EM-banded model and banded Ridge regression (la Tour et al., 2022;Nunez-Elizalde et al., 2019), where weight pruning (such as setting excessively shrunken regression weights to zero) would otherwise be necessary.

It is important to once again stress that the considered modeling framework may provide poor approximations to the true posterior distribution over the model weights. The hope is instead that the model yields reasonable out-of-sample predictions (Tipping, 2001), which we indeed found to be the case in several analyses. In encoding or decoding analyses, the emphasis is typically on out-of-sample predictive accuracy and point estimates of model parameters. Our motivation behind highlighting model weights in several of our simulations was to better illustrate model properties and highlight regression problems where group shrinkage can be relevant.

### 4.5 Over-fitting and cross-validation

Tuning hyperparameters using cross-validated estimates of expected prediction accuracy may reduce the risk of over-fitting. One benefit of tuning hyperparameters using cross-validation compared to empirical Bayes (without cross-validation) is that it optimizes an estimate of expected prediction accuracy which can lead to good generalization, especially in cases with many observations, high SNR, and independent training and validation splits. Moreover, such procedures can be highly useful for identifying model misspecifications. However, with limited and noisy data, the variance of the expected prediction accuracy estimate can be high. Tuning many free hyperparameters to optimize such an estimate can be associated with a considerable risk of over-fitting in these circumstances.

Cross-validation can similarly be used to tune hyperparameters of the EM-banded model. This may prove beneficial in some cases (compared to fixing model parameters*a priori*) and could potentially reduce the risk of over-fitting, but the same concerns apply here. In practice, rather than tuning all free hyperparameters of the EM-banded jointly, one may choose to introduce the constraint that onlyγis tuned, withγ=τ=η=κ=ϕ. This will practically make it more straightforward to fit EM-banded models across a range ofγparameters and tuneγto minimize expected prediction error on held-out data. Section 7 in theSupplementary Materialshows results from such procedures.

### 4.6 Other perspectives

We used the described modeling framework to fit encoding and decoding models trained on data from single subjects (i.e., single-subject analyses). The described framework could also be incorporated in “searchlight” decoding analyses (Kriegeskorte et al., 2006) where different subsets of features from multi-dimensional neural data recordings are used as predictors in single-subject or group-level decoding models. The framework could for example be relevant in studies that attempt to use one or more features extracted from multiple imaging modalities to predict clinical scores (Woo et al., 2017).

One appealing aspect of group shrinkage procedures is that they can allow for ranking importance of feature groups. This may aid interpretation of encoding analyses (la Tour et al., 2022;Nunez-Elizalde et al., 2019). In recent years, encoding models have been used increasingly to model neural responses with features extracted from task-optimized neural networks (Kell et al., 2018;Tuckute et al., 2023;Yamins et al., 2014;Yamins & DiCarlo, 2016). This typically involves very high-dimensional feature sets and hence encoding models where the number of predictors is much larger than the number of observations, that is,D≫M. Surprisingly, over-parameterized models may show good generalization performance even with vanishing explicit regularization in minimum-norm least squares estimators (Belkin et al., 2019;Hastie et al., 2022;Kobak et al., 2020). This behavior is clearly different from cases where explicit regularization is critical for performance. Yet, the ability to establish importance of sets of features can similarly be useful in these settings, and group shrinkage procedures may be one way of achieving this.

## 5 Conclusion

In this paper, we explored a framework for estimating banded-type regression models in encoding and decoding analyses. It offers a tool that can have relevance for specific modeling problems in computational neuroscience, complementing other related estimators. We used simulations and data examples to illustrate properties and limitations of the described model in comparison with Ridge estimators.

## Supplementary Material

Supplementary Material

## Data Availability

Code implementations in both Matlab (The MathWorks, Inc., Natick, MA, United States) and Python are available onhttps://github.com/safugl/embanded. The Python code utilizes libraries such as*Numpy*(Harris et al., 2020),*Scipy*(Virtanen et al., 2020), and*Pytorch*(Paszke et al., 2019). The public repository contains examples and runtime tests. EEG data and BOLD fMRI data used in this study have been presented previously (Broderick et al., 2018;Di Liberto et al., 2015;Nakai et al., 2021,^2022^) and are available onhttps://doi.org/10.5061/dryad.070jcandhttps://openneuro.org/datasets/ds003720, respectively.
