# Supplementary material for: Exploring an EM-algorithm for banded regression in computational neuroscience
Source: Imaging Neurosci (Camb). 2024 May 20;2:imag-2-00155. doi: 10.1162/imag_a_00155 (PMC12247579; doi:10.1162/imag_a_00155)
Supplement: Supplementary Material [file imag_a_00155-supp.pdf]

Exploring an EM-algorithm for banded regression in computational neuroscience

Supplementary Material

Søren A. Fuglsang[1,2,*], Kristoffer H. Madsen[1,3], Oula Puonti[1,4], Hartwig R. Siebner[1,5,6], and Jens Hjortkjær[1,2]

[1] *Danish Research Centre for Magnetic Resonance, Centre for Functional and Diagnostic Imaging and Research, Copenhagen University Hospital - Amager and Hvidovre, Copenhagen, Denmark.*
[2] *Hearing Systems Section, Department of Health Technology, Technical University of Denmark, Kgs. Lyngby, Denmark.*
[3] *Department of Applied Mathematics and Computer Science, Technical University of Denmark, Denmark, Kgs. Lyngby, Denmark.*
[4] *Martinos Center for Biomedical Imaging, Massachusetts General Hospital and Harvard Medical School, USA.*
[5] *Department of Neurology, Copenhagen University Hospital Bispebjerg, Copenhagen, Denmark.*
[6] *Institute for Clinical Medicine, Faculty of Health and Medical Sciences, University of Copenhagen, Copenhagen, Denmark.*

April 10, 2024

Contents

1 fMRI encoding analysis **2**
1.1 fMRI preprocessing . . . 2
1.2 Feature groups . . . 2
1.3 Regression . . . 2

2 EEG decoding analysis **3**
2.1 Preprocessing . . . 3
2.2 Regression . . . 3

3 Multiple output case **3**
3.1 Log-objective . . . 5

4 Simulating responses to acoustic and phonetic features **6**
4.1 Feature groups . . . 6
4.2 Alternative data-generating process . . . 6

5 Simulation with groups of correlated predictors **7**

6 Banded regression and hyperparameter tuning **8**

7 Out-of-sample prediction accuracy **10**

*sorenaf@drcmr.dk

1 fMRI encoding analysis

1.1 fMRI preprocessing

We used a publicly available BOLD fMRI dataset that has been described in (Nakai et al., 2022, 2021) which contains data acquired from five participants. Informed consent had been obtained from all participants prior to their participation, and procedures were approved by local ethics and safety committees, see Nakai et al. (2021, 2022). We considered a minimal BOLD fMRI preprocessing pipeline which involved motion correction (Friston et al., 1996), spatial smoothing (with a 3 mm FWHM Gaussian kernel), and brain extraction. These processing steps utilized SPM 12.6685 (Penny et al., 2011) and FSL 6.0.1 (Jenkinson et al., 2012; Smith et al., 2004). Part of the motivation for incorporating spatial smoothing was to improve signal-to-noise ratio, but also that susceptibility-induced distortions rendered it difficult to adequately align volumes across functional runs. Six motion regressors (translations in x, y, and z directions and rotations about x, y, and z) were extracted for each subject and functional run. These were used to create a set of nuisance regressors that further included a cosine basis set from a one-dimensional discrete cosine transform. Time courses from each voxel were deflated using this set of regressors. We defined the average response to a given sound stimulus clip as the average of these residualized voxel time courses from 6 s to 16.5 s after stimulus onset. This was a pragmatic choice and it means that only a subset of each stimulus clip contributes to the estimated "average" response (while minimizing contamination from the previous stimulus clip). For the last stimulus clip, we averaged from 6 s after stimulus onset to the last acquired volume. The initial 15 s of 'dummy' images in each functional run were omitted from subsequent analyses in both test and training runs. All analyses were conducted in native subject space. For visualization purposes, we mapped the resulting maps of predictive accuracy to a Montreal Neurological Institute (MNI) template. This was achieved using tools available with Advanced Normalization Tools (ANTs) (Avants et al., 2009). Visualization of results relied on scientific computing software in Python, and utilized libraries such as *Nilearn* (Abraham et al., 2014), *Seaborn* (Waskom, 2021), *Numpy* (Harris et al., 2020), and *Matplotlib* (Hunter, 2007).

1.2 Feature groups

We defined three features groups, two of which were related to the audio stimuli. The first, F_1, was a genre-label dummy-coded feature that indicated which of 10 genres each stimulus clip had been assigned to by Tzanetakis and Cook (2002). The second, F_2, was a time-averaged "spectrogram" representation. To generate this representation, each stimulus clip was passed through a 32-channel gammatone filterbank (Slaney et al., 1993) with filter centre frequencies spaced between 50 Hz and 11050 Hz (Glasberg and Moore, 1990). Envelopes were extracted from the output of each filter via the absolute value of the Hilbert transform and subsequently power-law compressed with a compression factor of $c = 0.3$. We defined the spectrogram representation of each stimulus clip as the temporal average of these features from 2 s to 11 s after stimulus onset. Finally, we considered a third feature group, F_3, which contained 400 stimulus-irrelevant noise predictors. These predictors were simulated for each stimulus block as a random vector from a multivariate Gaussian distribution with zero mean and covariance $C = \exp(-0.3|k-i|^2), k = 1, ..., 400, i = 1...400$.

1.3 Regression

Separate regression models were fit to data from each voxel. Predictors and the voxel response time courses were standardized. For each voxel, we fit models to data from the 12 training runs and evaluated predictive accuracy on data from held-out test runs. For the EM-banded estimator, we defined distinct λ_j parameters to each of the three feature groups and defined that $\tau = \eta = \kappa = \phi = 10^{-4}$. For the Ridge estimator, we considered a 5-fold cross-validation procedure to inform the selection of α from data from the 12 training runs. A range of possible α values over a grid of 50 logarithmically spaced values between 10^{-5} and 10^{10} were considered. The chosen α parameter was the parameter that minimized the average mean square error between model predictions and target responses in held-out folds. Prediction accuracies on the test set were quantified in two ways. First, we computed the Pearson correlation coefficient between predicted responses in test runs and the target responses. Second, for each voxel time course, we computed R^2 defined as follows:

$$R^2 = 1 - \frac{\sum_i (y_i - \hat{y}_i)^2}{\sum_i (y_i - \overline{y})^2}, \tag{1}$$

where $\hat{y}_i$ is the predicted response, where y_i is the target response, and where $\overline{y}$ is the mean response.

2 EEG decoding analysis

2.1 Preprocessing

We used a publicly available EEG dataset (https://doi.org/10.5061/dryad.070jc) which includes EEG data from 19 participants that each undertook 20 experimental trials in which they listened to speech stimuli (Broderick et al., 2019; Di Liberto et al., 2015). All procedures were undertaken in accordance with the Declaration of Helsinki and were approved by local ethics committees, see Broderick et al. (2019); Di Liberto et al. (2015). The dataset includes a 'broadband' envelope representation of the speech stimulus in each experimental trial. The first-order temporal difference of this envelope representation was computed and the output was then half-wave rectified (Synigal et al., 2020; Hertrich et al., 2012; Daube et al., 2019), centered and band-pass filtered between 1 Hz and 8 Hz using a second-order Butterworth filter. Zero-phase filtering was achieved by filtering in the forward and reverse directions. The dataset includes EEG data from each experimental trial sampled at 128 Hz. These data were re-referenced to the common average, detrended (second-degree polynomial trends), and low pass filtered at 40 Hz using a fourth-order zero-phase Butterworth filter. We defined two EEG feature groups from these preprocessed data: low-frequency (LF) features and higher-frequency (HF) features. For the LF features, we filtered the data between 1 Hz and 8 Hz using the same filters as those applied to the audio envelope representations. For the HF features, we filtered the data between 25 Hz and 35 Hz using second-order zero-phase Butterworth filters and then computed the absolute value of the Hilbert transform from each electrode time course. These time courses were subsequently filtered between 1 Hz and 8 Hz using again the same filters as those applied to the audio envelope representations. Finally, the EEG and audio features were downsampled to 40 Hz to reduce computation time. We augmented EEG features from each channel with a set of time lags by concatenating the time-lagged features side-by-side so as to absorb temporal mismatches between stimulus features and EEG features in the subsequent regression (De Cheveigné et al., 2021). The time lags ranged from -250 ms to 0 ms. We focused on data from 10 s post-trial onset to approximately 160 s post-trial onset for both EEG and audio features.

2.2 Regression

We focused on decoding models that utilized time-lagged LF and HF EEG features to predict the speech envelope feature. We used a leave-one-trial-out outer cross-validation approach, fitting models to data from all trials except one and evaluating prediction accuracy on the held-out trial. This procedure was repeated for all trials. For the EM-banded model, we grouped predictors into $j = 1, ..., 256$ sets, each consisting of multiple time-lagged versions of either an LF or HF feature from a single electrode. During cross-validation, we computed the means and standard deviations of the training data. Subsequently, we transformed both the training data and the held-out data by subtracting these mean values and dividing by these standard deviations. This was done separately for each predictor and for the target variable. The scale of features extracted from single-trial EEG can vary dramatically across subjects and electrodes, and it can thus be convenient to standardize the data when focusing on default prior distributions on model weights and default initialization schemes.

For the EM-banded model, we fixed hyperparameters related to the Inverse-Gamma priors to $\gamma = 10^{-4}$, and further encouraged smoothness on temporal filters for each predictor group by defining that $h_j = 0.5$ for all $j = 1, ..., 256$ groups (with h_j defined as in the main text). For the Ridge estimator, we further considered an inner 5-fold cross-validation procedure. The α parameter was tuned to maximize average correlation between predictions and target envelope features in held-out inner folds. We considered α values ranging from 10^{-5} to 10^{10} in 50 logarithmically spaced steps.

Prediction accuracies were defined as the Pearson correlation between model predictions and target envelope feature in held-out test trials. The correlation coefficients were averaged across trials after first applying the Fisher-Z transform and the average was inverse-transformed using the inverse of the Fisher-Z transform. Visualization of results in Python utilized libraries such as *MNE-Python* (Gramfort et al., 2013), *matplotlib* (Hunter, 2007) and *seaborn* (Waskom, 2021).

3 Multiple output case

Here we formulate an EM-banded model for the regression scenario with multiple outcome variables and a design matrix X that is shared for all outcome variables. Let $Y \in \mathrm{R}^{M \times P}$ be a matrix with $p = 1, ..., P$ outcome variables stacked column-wise. In this case, the regression problem can be written in matrix notation as follows:

$$Y = XW + E. \tag{2}$$

Here, $X \in \mathrm{R}^{M \times D}$ denotes the design matrix, $W \in \mathrm{R}^{D \times P}$ denotes the weights and $E \in \mathrm{R}^{M \times P}$ denotes error terms. We will once again assume that the design matrix X can be partitioned into J meaningful groups such that $X = [F_1, ..., F_j, ..., F_J]$, where each group F_j has one or more predictors stacked column-wise. Let D_j denote the number of predictors in the j-th group such that the total number of predictors equals $D = \sum_j D_j$. Further, let $\beta_{j,p} \in \mathrm{R}^{D_j \times 1}$ be a column vector that contains D_j weights associated with predictor group j for outcome variable p. We partition W and Λ as follows:

$$W = \begin{bmatrix} \beta_{1,1} & \beta_{1,2} & \dots & \beta_{1,P} \\ \vdots & \vdots & \ddots & \vdots \\ \beta_{J,1} & \beta_{J,2} & \dots & \beta_{J,P} \end{bmatrix} \tag{3}$$

$$\Lambda \equiv \begin{bmatrix} \lambda_1 I_{D_1} & \dots & 0 \\ \vdots & \ddots & \vdots \\ 0 & \dots & \lambda_J I_{D_j} \end{bmatrix} \begin{bmatrix} \Omega_1 & \dots & 0 \\ \vdots & \ddots & \vdots \\ 0 & \dots & \Omega_J \end{bmatrix}. \tag{4}$$

Each Ω_j block has size $D_j \times D_j$ and follows the definition in the main text. We will henceforth represent vectorized versions of Y and W as y and w respectively, defined as follows:

$$w \equiv \mathrm{vec}(W) = \left[\beta_{1,1}^\top, ..., \beta_{J,1}^\top, \beta_{1,2}^\top, ..., \beta_{J,2}^\top, ..., \beta_{1,P}^\top, ..., \beta_{J,P}^\top\right]^\top \tag{5}$$

$$y \equiv \mathrm{vec}(Y) \tag{6}$$

$$\tag{7}$$

where $\mathrm{vec}(Y)$ denotes the vec-operator applied on Y. Additionally, let $H \equiv I_P \otimes X$ where $\otimes$ denotes Kronecker product. To simplify notation, we will again let λ denote a set of $\{\lambda_j\}_{j=1}^J$ parameters and not highlight dependence on terms that remain fixed. We will assume that each outcome variable has been scaled in a way that justifies the following model:

$$p(y|H, w, \nu) = \mathcal{N}(y|Hw, \nu I_{M \cdot P}), \tag{8}$$

$$p(w|\lambda, \eta, \tau) = \mathcal{N}(w|0, I_P \otimes \Lambda), \tag{9}$$

$$p(\lambda_j|\eta, \tau) = \text{Inv-Gamma}\left(\lambda_j \,|\eta, \tau\right), \quad j = 1, ..., J \tag{10}$$

$$p(\nu|\phi, \kappa) = \text{Inv-Gamma}\left(\nu \,|\phi, \kappa\right), \tag{11}$$

Here, $I_{M \cdot P}$ denotes an identity matrix of size $(M \cdot P) \times (M \cdot P)$. Letting $\Sigma \equiv \left(\Lambda^{-1} + \nu^{-1} X^\top X\right)^{-1}$ and $B \equiv \nu^{-1} \Sigma X^\top Y$ we see that:

$$p(w|y, H, \lambda, \nu) = \mathcal{N}(w|\mu, S), \tag{12}$$

$$S = \left((I_P \otimes \Lambda)^{-1} + \nu^{-1} H^T H\right)^{-1} = I_P \otimes \Sigma \tag{13}$$

$$\mu = \nu^{-1} S H^\top y = \mathrm{vec}\left(B\right) \tag{14}$$

We let $\tilde{B}$ and $\tilde{\Sigma}$ denote B respectively Σ estimated for a given set of λ and ν. We subsequently partition the matrix $\tilde{B}$ as follows:

$$\tilde{B} = \begin{bmatrix} \tilde{\mu}_{1,1} & \tilde{\mu}_{1,2} & \dots & \tilde{\mu}_{1,P} \\ \vdots & \vdots & \ddots & \vdots \\ \tilde{\mu}_{J,1} & \tilde{\mu}_{J,2} & \dots & \tilde{\mu}_{J,P} \end{bmatrix}, \tag{15}$$

where $\tilde{\mu}_{j,p}$ denotes a column vector with elements associated with predictor group j for outcome p. We let $\tilde{\Sigma}_j$ denote a block of size $D_j \times D_j$ along the diagonal in $\tilde{\Sigma}$ associated with group j. We follow the same procedure as in the main text and find the following closed-form update rules:

$$\lambda_j^{(k+1)} = \frac{\sum_{p=1}^{P}\left[\tilde{\mu}_{j,p}^\top \Omega_j^{-1}\tilde{\mu}_{j,p} + \mathrm{Tr}(\Omega_j^{-1}\tilde{\Sigma}_j)\right] + 2\tau}{P \cdot D_j + 2\eta + 2} \tag{16}$$

$$\nu^{(k+1)} = \frac{\mathrm{Tr}\left((Y - X\tilde{B})^\top (Y - X\tilde{B})\right) + P \cdot \mathrm{Tr}(X^\top X\tilde{\Sigma}) + 2\kappa}{P \cdot M + 2 + 2\phi} \tag{17}$$

To simplify this expression further, we define that $\tilde{B}_j$ is a block in $\tilde{B}$ of size $D_j \times P$ with elements associated with group j, such that $\tilde{B}_j = [\tilde{\mu}_{j,1}, \tilde{\mu}_{j,2}, ..., \tilde{\mu}_{j,P}]$. We can now write:

$$\lambda_j^{(k+1)} = \frac{\mathrm{Tr}\left(\tilde{B}_j^\top \Omega_j^{-1}\tilde{B}_j\right) + P \cdot \mathrm{Tr}(\Omega_j^{-1}\tilde{\Sigma}_j) + 2\tau}{P \cdot D_j + 2\eta + 2} \tag{18}$$

$$\nu^{(k+1)} = \frac{\mathrm{Tr}\left((Y - X\tilde{B})^\top (Y - X\tilde{B})\right) + P \cdot \mathrm{Tr}(X^\top X\tilde{\Sigma}) + 2\kappa}{P \cdot M + 2 + 2\phi} \tag{19}$$

It should be clear that the above model is similar to the model described in the main text when $P = 1$. Note that it can be relevant to compute $X^\top X$ and $X^\top Y$ and avoid computing these terms multiple times. It can also be relevant to use the cyclic property of the trace in some scenarios (e.g., when $P > D$). Additionally, it can be relevant to make use of the Woodbury matrix identity (Murphy, 2012) and define $\Sigma = \Lambda - \Lambda X^\top(\nu I_M + X\Lambda X^\top)^{-1}X\Lambda$ when $D \gg M$.

3.1 Log-objective

We recall that our goal is to maximize the marginal posterior density $p(\lambda, \nu | y, H)$ which is equivalent to maximization of $p(\lambda)p(\nu)p(y|\lambda, \nu, H)$. Here, the logarithm of $p(y|\lambda, \nu, H)$ takes the form:

$$\ln p(y|\lambda, \nu, H) = -\frac{M \cdot P}{2}\ln 2\pi - \frac{1}{2}y^\top C^{-1} y - \frac{1}{2}\ln|C|, \tag{20}$$

with $C = \nu I_{M \cdot P} + H(I_P \otimes \Lambda)H^\top$. The matrix C has size $(M \cdot P) \times (M \cdot P)$ which could be problematic if one were to directly evaluate Eq. 20 in a regression problem with many outcome variables. We follow Tipping (2001) and simplify this expression using the Woodbury matrix identity and the matrix determinant identity. In this case, $\ln|C|$ can be simplified and written as follows:

$$\begin{aligned}
\ln|C| &= -\ln|S| + P\ln|\Lambda| + P \cdot M\log(\nu) \\
&= P \cdot \left(-\ln|\Sigma| + \sum_{j=1}^{J}\ln|\Omega_j| + \sum_{j=1}^{J} D_j \ln\lambda_j + M\log(\nu)\right),
\end{aligned}$$

Notice that Σ has size $D \times D$. One can similarly simplify $y^\top C^{-1} y$ and express it in terms of B:

$$\begin{aligned}
y^\top C^{-1} y &= y^\top\left(\nu^{-1} I_{P \cdot M} - \nu^{-1} HSH^\top \nu^{-1}\right) y \\
&= \nu^{-1} y^\top (y - H\mu) \\
&= \nu^{-1} y^\top (y - \mathrm{vec}(XB))
\end{aligned}$$

We can now write the expression in Eq. 20 as follows:

$$\ln p(y|\lambda, \nu, X) = -\frac{M \cdot P}{2}\ln 2\pi - \frac{1}{2\nu}y^\top(y - \mathrm{vec}(XB)) - \frac{P}{2}\left(-\ln|\Sigma| + \sum_{j=1}^{J}\ln|\Omega_j| + \sum_{j=1}^{J} D_j\ln\lambda_j + M\log(\nu)\right)$$

This is usually fast to compute even when there is a high number of outcome variables and hence dimensions in C. Our implementation of the algorithm incorporates this expression when computing the logarithm of the objective function

(hereafter referred to as log-objective) at each iteration. Ignoring terms that remain fixed during optimization with a fixed set of hyperprior parameters, we express the log-objective Δ as follows:

$$\Delta \propto \ln p(y|\lambda, \nu, X) - (1+\phi)\ln\nu - \frac{\kappa}{\nu} - \sum_{j=1}^{J}(1+\eta)\ln\lambda_j - \sum_{j=1}^{J}\frac{\tau}{\lambda_j}$$

The implementation makes it possible to define a convergence criterion based on increases in the log-objective, such that the algorithm will terminate if increases in the log-objective are below some user-defined tolerance. We leave it up to future studies to explore alternative (faster) approaches to maximizing the log-objective.

4 Simulating responses to acoustic and phonetic features

4.1 Feature groups

Data from the *The DARPA TIMIT Acoustic-Phonetic Continuous Speech Corpus* (TIMIT) (Garofolo et al., 1993) were used for this simulation example. We utilized sentences spoken by 190 speakers for this example, with each speaker delivering ten sentences. Audio waveforms were root mean square normalized. Auditory spectrogram features were extracted from each audio waveform. To generate these features, we passed each waveform through a 32-channel gammatone filterbank (Slaney et al., 1993) with filter centre frequencies spaced between 50 Hz and 8000 Hz. Envelopes were extracted from the output of each filter via the absolute value of the Hilbert transform and subsequently power-law compressed with a compression factor of $c = 0.3$. The envelopes were lowpass filtered at 64 Hz (second-order zero-phase Butterworth filter) and downsampled to 128 Hz. For the phonetic features, we defined boxcar regressors based on time-aligned phonetic labels available from each sentence (Garofolo et al., 1993; Zue and Seneff, 1996). Specifically, we constructed six boxcar regressors flagging time periods where the annotation indicated the occurrence of the following categories: stops, affricatives, fricatives, nasals, semivowels and glides, and vowels. We define these boxcar regressors as phonetic features in this simulation. It should be stressed that our goal is not to make statements about the coding of phonetic features, but rather to illustrate properties of regularized estimators when considering such stimulus feature sets. Spectrogram features and phonetic features were extracted from each sentence. Next, we created multiple time-lagged versions of each predictor in these feature sets for the time lags: 0, 1, ..., 38. The first 39 samples of features extracted from each sentence were subsequently discarded. This procedure was repeated for all sentences and the features were stacked row-wise and subsequently standardized. We let F_1 denote time-lagged spectrogram features and F_2 denote time-lagged phonetic features. The procedure resulted in a total of 661633 samples, corresponding to approximately 86 minutes of data. The feature group F_1 has 1248 columns and the feature group F_2 has 234 columns

4.2 Alternative data-generating process

Simulation 4 in the main text assumes that the response variable only contains a mixed version of F_1, but not of F_2. For completeness, Figure 1 depicts results of a similar simulation where the response is simulated as $y = F_2 w_2 + \epsilon$, such that only the phonetic features are assumed to affect the response. Ridge models and EM-banded models are again fit to the data using both feature sets. The simulated "target" weights w_2 are shown in Figure 1A.

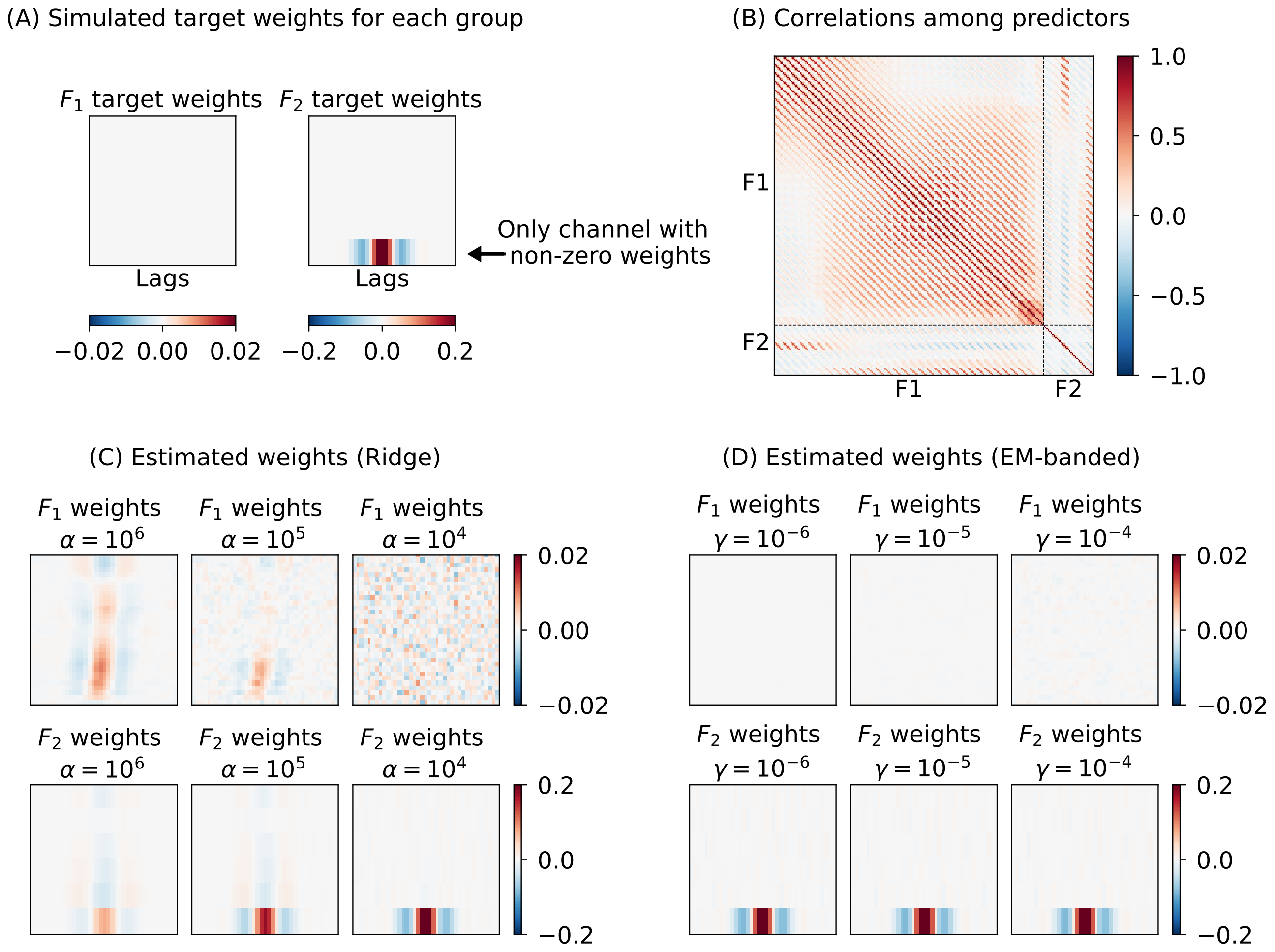

Figure 1: (A) Target weights associated with each of the two feature sets. Here, only phonetic features are simulated to affect the response. (B) Matrix of Pearson correlation coefficients between predictors. Dashed lines are used to visualize predictor groups, F_1 and F_2. (C) Weights estimated with Ridge estimator for each of the two groups. (D) Weights estimated with the EM-banded estimator for each of the two groups. Ridge estimators with α set to 10^6, 10^5 and 10^4 are shown. EM-banded estimators were considered with γ set to 10^{-6}, 10^{-5} and 10^{-4}

5 Simulation with groups of correlated predictors

Here, we consider a simulation in which we simulate two groups of predictors, F_1 and F_2. Each of these two feature groups has 128 dimensions and 2048 rows. We assume $y = F_1 w_1 + \epsilon$ and that only F_1 affects the outcome variable, y. The target weights w_1 are shown in Figure 2. As previously, we assume that ϵ is drawn from a Gaussian distribution. Each row in $[F_1, F_2]$ is drawn from a multivariate Gaussian with zero mean and a covariance C defined as follows:

$$C = \begin{bmatrix} I_{128} & 0.3 \cdot I_{128} \\ 0.3 \cdot I_{128} & I_{128} \end{bmatrix}$$

This simulates correlations among predictors associated with the different groups. The SNR is 0 dB. We again compare Ridge regression model fits with EM-banded model fits using a set of fixed hyperparameters. The estimated weights for the Ridge and EM-banded model are shown in Figure 2. The EM-banded estimator excessively shrinks the weights associated with F_2 (as desired) when γ attains a low value. In all cases, the EM-banded model accurately recovers the weights associated with F_1. The Ridge estimator, on the other hand, tends to pull correlated weights towards each other when α attains a high value (in this case when $\alpha = 1000$ and $\alpha = 10000$). This has the undesired consequence that the weights associated with F_2 tend to resemble the weights associated with F_1 more. This could potentially lead to misinterpretation of the weights associated with F_2.

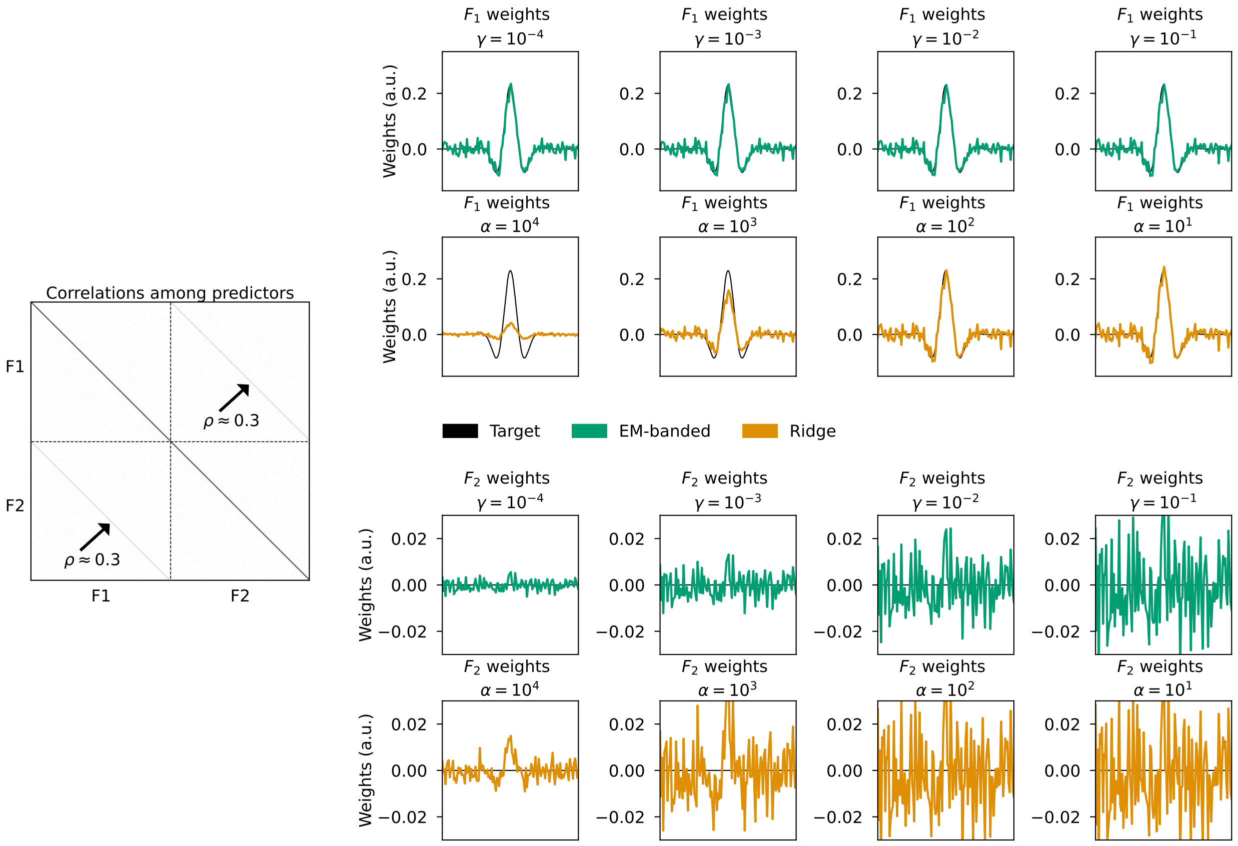

Figure 2: Simulation 6 illustrating behavior of the Ridge estimator and the EM-banded estimators when there are correlations among predictors that have been divided into two groups, F_1 and F_2. Left middle panel: Matrix of Pearson correlation coefficients between predictors. Dashed lines are used to visualize predictor groups, F_1 and F_2. Right: Weights estimated with Ridge estimator and with the EM-banded estimator. The top two rows indicate weights associated with F_1. The bottom two rows indicate weights associated with F_2. The target weights are shown in black.

6 Banded regression and hyperparameter tuning

Banded Ridge regression avoids making explicit assumptions about the prior distribution of hyperparameters and instead uses cross-validation to tune hyperparameters. Using cross-validation to tune regularization strength and optimize predictive accuracy on held-out data may be a strategy to facilitate feature selection and interpretability of model parameters. However, tuning multiple free hyperparameters using cross-validation can also lead to spurious excessive shrinkage of weights associated with a given feature band, e.g., if the variance of the expected prediction accuracy estimate is high, complicating the interpretation of model parameters.

To illustrate this point, the following simulation shows an unfavorable SNR situation similar to Simulation 5 in the main text, but here focuses on a banded Ridge regression estimator. As in Simulation 5, we simulate two predictors, F_1 and F_2, and a response variable $y = F_1 w_1 + F_2 w_2 + \epsilon$. The predictors, as well as the noise term ϵ, are drawn from Gaussian distributions, and the number of samples is 512. The target weights are fixed to non-zero values and the SNR is approximately -20 dB. We now focus on the following estimator:

$$w = \left(\begin{bmatrix} F_1 & F_2 \end{bmatrix}^T \begin{bmatrix} F_1 & F_2 \end{bmatrix} + L\right)^{-1} \begin{bmatrix} F_1 & F_2 \end{bmatrix}^T y,$$

$$L = \begin{bmatrix} \alpha_1 & 0 \\ 0 & \alpha_2 \end{bmatrix}.$$

This estimator is fit to the training data for different values of α_1 and α_2. When $\alpha_1 = \alpha_2$, this corresponds to a

standard Ridge estimator.

Next, we simulate five independent validation sets, each with 512 samples. The simulation process for predictors, noise, and target variables mirrors that of the training set. For example, for the first validation set, we simulate two predictors $\hat{F}_1$ and $\hat{F}_2$ and response variable $\hat{y} = \hat{F}_1 w_1 + \hat{F}_2 w_2 + \hat{\epsilon}$, where $\hat{F}_1$, $\hat{F}_2$, and $\hat{\epsilon}$ are drawn from Gaussian distributions, and where w_1 and w_2 are the same target weights as for the training set. For each validation set, we evaluate how well the model predicts the target variable $\hat{y}$ for different values of α_1 and α_2.

If one were to select hyperparameters, α_1 and α_2, to minimize MSE (or maximize correlation), then this may lead to excessive shrinkage applied to one of the two weights, to neither of the weights, or to both, depending on the validation set. Moreover, the level of regularization declared to each weight may strongly depend on performance metric used for parameter tuning (e.g., MSE). Using similar tuning procedures for the Ridge estimator may also result in different amounts of shrinkage depending on the specific validation set, but in this case, (nearly) the same amount of shrinkage would be applied to both weights (hence having little impact on Pearson correlation coefficients).

These simulations are highly stylized and mainly serve to assist intuition about how interpretability of model parameters in banded regression with multiple 'bands' similarly can be complicated, also when regularization hyperparameters are tuned using cross-validation. This once again highlights the relevance of exploring various models to better understand whether model properties are inappropriate for a given problem.

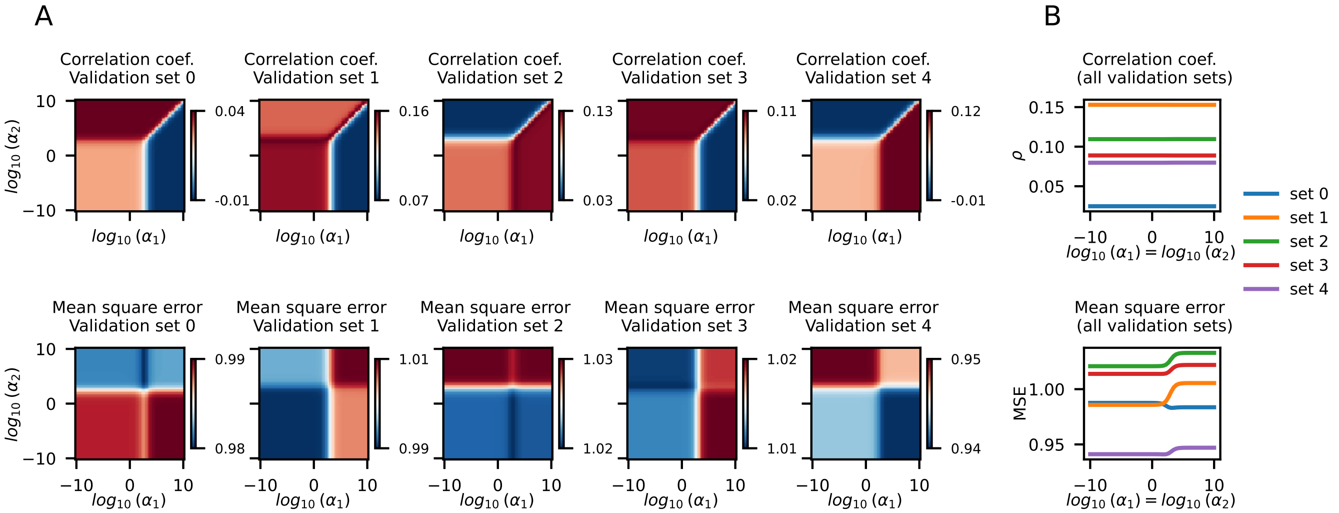

Figure 3: Results from a simulation with a single training set and multiple validation sets. (A) Results from analyses across a range of α_1 and α_2 values. (B) Results from analyses with $\alpha_1 = \alpha_2$. Each line in panel (B) represents data from a single validation set. Top row: Pearson correlation coefficient between model predictions and target variable in each validation set. Botton row: Mean square error between model predictions and target variable in each validation set.

7 Out-of-sample prediction accuracy

Simulations 1-3 in the main text illustrate weights estimated by the EM-banded model in different simulation scenarios. Here, we explore how well the models in these simulations predict novel data with different hyperparameter settings. For each of these three simulations, we simulate validation data using the same data-generating processes as for the training data. The same target weights are considered for both the training data and the validation data. For instance, in Simulation 1, we simulate three groups of predictors ($\hat{F}_1$, $\hat{F}_2$, and $\hat{F}_3$, each with 64 predictors) and simulate a response variable $\hat{y}$ as $\hat{y} = \hat{F}_1 w_1 + \hat{F}_3 w_3 + \hat{\epsilon}$, where w_1 and w_3 represent the same target weights as described in the main text, and where $\hat{\epsilon}$ indicates Gaussian noise. We now fit EM-banded models to the training data and attempt to predict $\hat{y}$ given the model and given $\hat{F}_1$, $\hat{F}_2$, and $\hat{F}_3$. We evaluate predictive accuracy in two ways: using mean square error between model predictions and $\hat{y}$, and using the Pearson correlation coefficient between model predictions and $\hat{y}$. This procedure is repeated for all three simulations and for EM-banded models fit with different values of τ, η, κ, and ϕ. Additionally, the procedure is repeated for Ridge regression models. For completeness, we also illustrate prediction accuracy across a range of a and b parameters where we define:

$$p(\lambda_j|\eta,\tau) = \text{Inv-Gamma}\left(\lambda_j\,|\eta,\tau\,\right) = \text{Inv-Gamma}\left(\lambda_j \,\middle|\, \frac{a}{2}, \frac{a \cdot b}{2}\right), \quad j = 1, ..., J$$

Results from all these analyses are shown in Figures 4, 5, and 6 for Simulations 1, 2, and 3 respectively. Notice that these figures also show results from analyses where we introduce the constraint that τ, η, κ, and ϕ all should equal the same value, γ. We observe that prediction accuracy tends to plateau as γ attains low values in all these simulations (see Fig. 4C, Fig. 5C, and Fig. 6C).

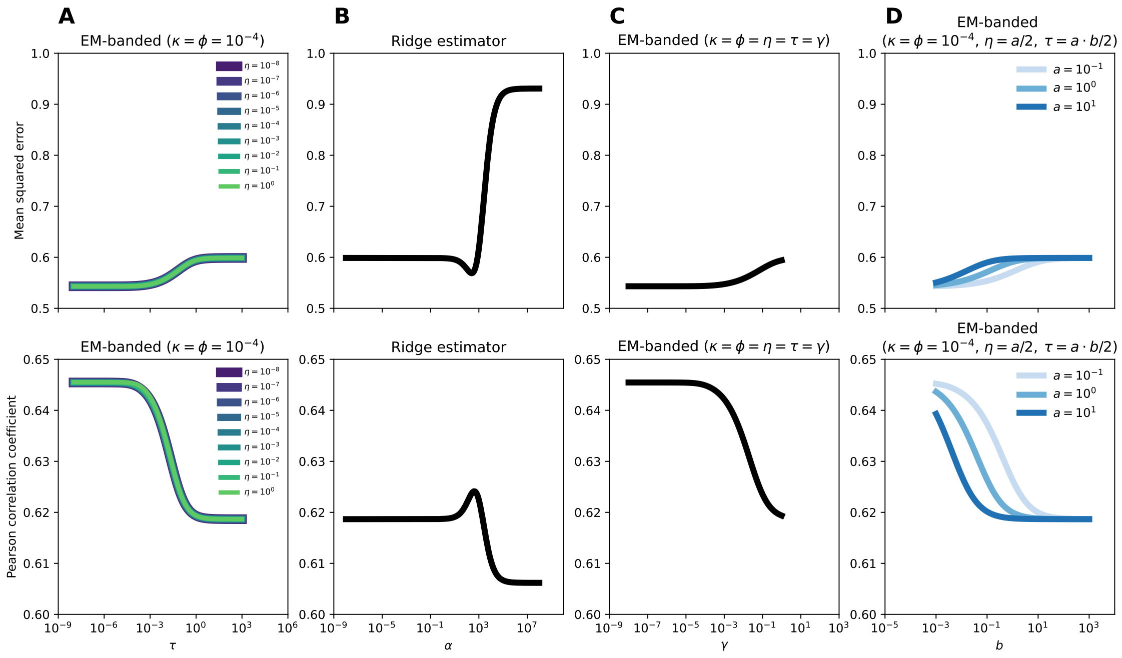

Figure 4: Simulation 1. Top row: Mean square error between model predictions and target variable. Bottom row: Pearson correlation coefficient between model predictions and target variable. (A): results obtained with EM-banded models fit with different values for τ and η, but with κ and ϕ kept fixed to a low value of 10^{-4}. (B): results obtained with the Ridge model across a range of α parameters. (C): results obtained with EM-banded models fit with different values for γ where $\tau = \eta = \kappa = \phi = \gamma$. (D): results obtained with EM-banded models fit with different values for a and b where $\eta = a/2$ and $\tau = a \cdot b/2$, and where κ and ϕ are kept fixed to 10^{-4}.

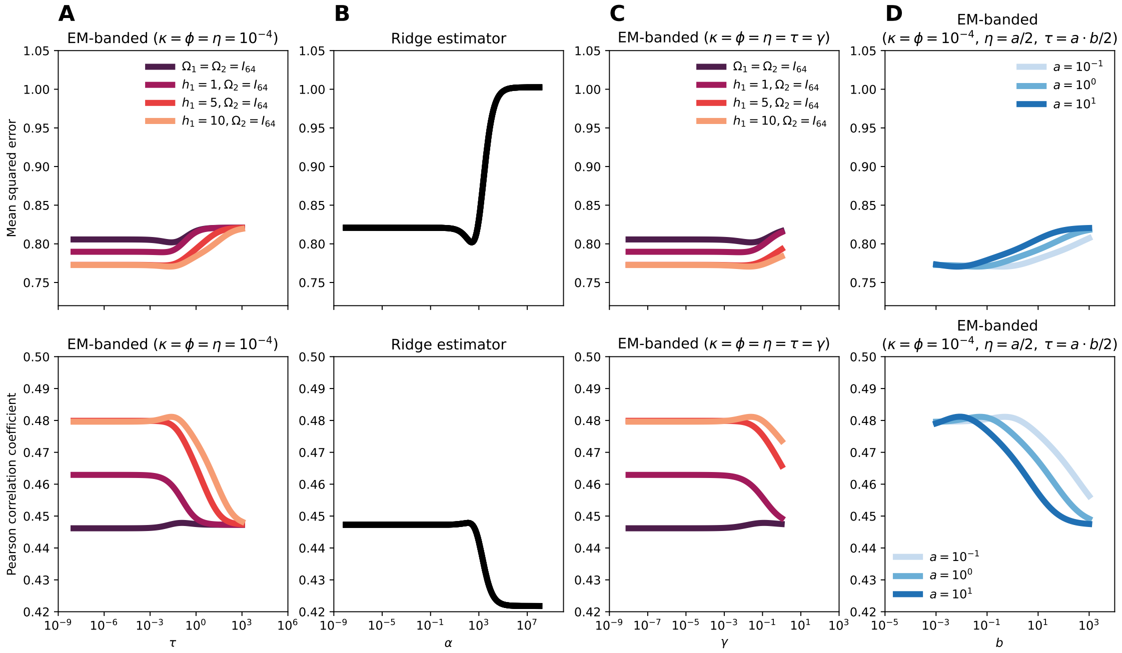

Figure 5: Simulation 2. Top row: Mean square error between model predictions and target variable. Bottom row: Pearson correlation coefficient between model predictions and target variable. (A): results obtained with EM-banded models fit with different values for h_1 and τ, but with κ, η and ϕ kept fixed to a low value of 10^{-4}. (B): results obtained with the Ridge model across a range of α parameters. (C): results obtained with EM-banded models fit with different values for γ and h_1 where $\tau = \eta = \kappa = \phi = \gamma$. (D): results obtained with EM-banded models fit with different values for a and b where $\eta = a/2$ and $\tau = a \cdot b/2$. Here, we kept κ and ϕ fixed to 10^{-4} and further kept h_1 fixed to 10.

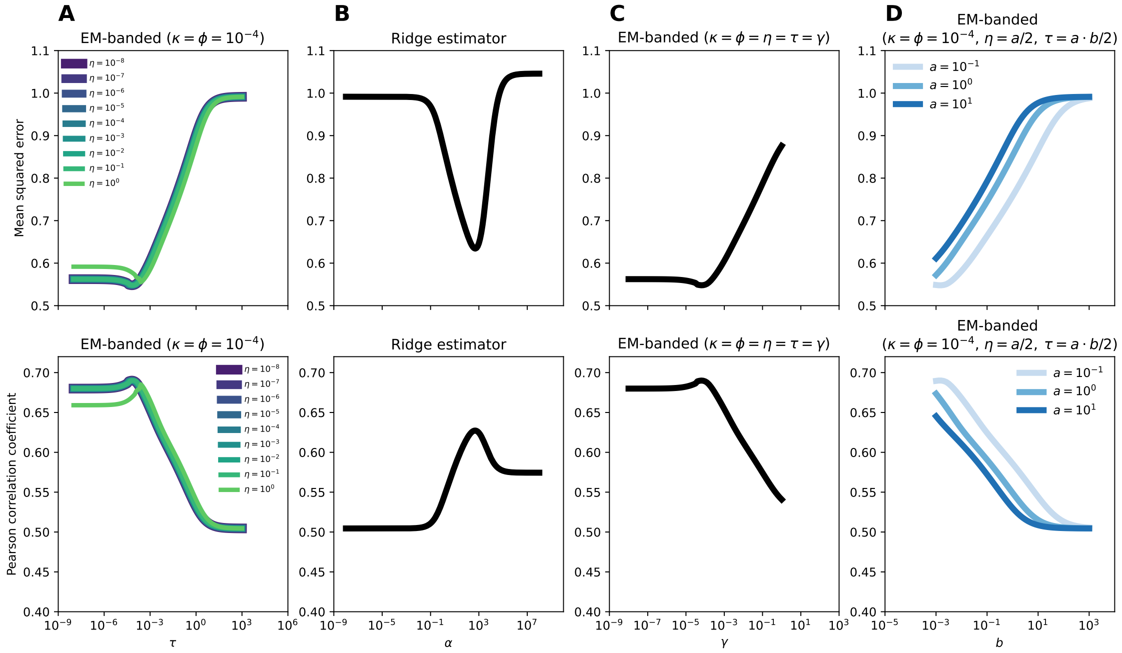

Figure 6: Simulation 3. Top row: Mean square error between model predictions and target variable. Bottom row: Pearson correlation coefficient between model predictions and target variable. (A): results obtained with EM-banded models fit with different values for τ and η, but with κ and ϕ kept fixed to a low value of 10^{-4}. (B): results obtained with the Ridge model across a range of α parameters. (C): results obtained with EM-banded models fit with different values for γ where $\tau = \eta = \kappa = \phi = \gamma$. (D): results obtained with EM-banded models fit with different values for a and b where $\eta = a/2$ and $\tau = a \cdot b/2$, and where κ and ϕ are kept fixed to 10^{-4}.

References

Abraham, A., Pedregosa, F., Eickenberg, M., Gervais, P., Mueller, A., Kossaifi, J., Gramfort, A., Thirion, B., and Varoquaux, G. (2014). Machine learning for neuroimaging with scikit-learn. *Frontiers in neuroinformatics*, page 14.

Avants, B. B., Tustison, N., Song, G., et al. (2009). Advanced normalization tools (ants). *Insight j*, 2(365):1–35.

Broderick, M. P., Anderson, A. J., and Lalor, E. C. (2019). Semantic context enhances the early auditory encoding of natural speech. *Journal of Neuroscience*, 39(38):7564–7575.

Daube, C., Ince, R. A., and Gross, J. (2019). Simple acoustic features can explain phoneme-based predictions of cortical responses to speech. *Current Biology*, 29(12):1924–1937.

De Cheveigné, A., Slaney, M., Fuglsang, S. A., and Hjortkjaer, J. (2021). Auditory stimulus-response modeling with a match-mismatch task. *Journal of Neural Engineering*, 18(4):046040.

Di Liberto, G. M., O'Sullivan, J. A., and Lalor, E. C. (2015). Low-frequency cortical entrainment to speech reflects phoneme-level processing. *Current Biology*, 25(19):2457–2465.

Friston, K. J., Williams, S., Howard, R., Frackowiak, R. S., and Turner, R. (1996). Movement-related effects in fmri time-series. *Magnetic resonance in medicine*, 35(3):346–355.

Garofolo, J. S., Lamel, L. F., Fisher, W. M., Fiscus, J. G., and Pallett, D. S. (1993). Darpa timit acoustic-phonetic continous speech corpus cd-rom. nist speech disc 1-1.1. *NASA STI/Recon technical report n*, 93:27403.

Glasberg, B. R. and Moore, B. C. (1990). Derivation of auditory filter shapes from notched-noise data. *Hearing research*, 47(1-2):103–138.

Gramfort, A., Luessi, M., Larson, E., Engemann, D. A., Strohmeier, D., Brodbeck, C., Goj, R., Jas, M., Brooks, T., Parkkonen, L., et al. (2013). Meg and eeg data analysis with mne-python. *Frontiers in neuroscience*, page 267.

Harris, C. R., Millman, K. J., van der Walt, S. J., Gommers, R., Virtanen, P., Cournapeau, D., Wieser, E., Taylor, J., Berg, S., Smith, N. J., Kern, R., Picus, M., Hoyer, S., van Kerkwijk, M. H., Brett, M., Haldane, A., del Río, J. F., Wiebe, M., Peterson, P., Gérard-Marchant, P., Sheppard, K., Reddy, T., Weckesser, W., Abbasi, H., Gohlke, C., and Oliphant, T. E. (2020). Array programming with NumPy. *Nature*, 585(7825):357–362.

Hertrich, I., Dietrich, S., Trouvain, J., Moos, A., and Ackermann, H. (2012). Magnetic brain activity phase-locked to the envelope, the syllable onsets, and the fundamental frequency of a perceived speech signal. *Psychophysiology*, 49(3):322–334.

Hunter, J. D. (2007). Matplotlib: A 2d graphics environment. *Computing in science & engineering*, 9(03):90–95.

Jenkinson, M., Beckmann, C. F., Behrens, T. E., Woolrich, M. W., and Smith, S. M. (2012). Fsl. *Neuroimage*, 62(2):782–790.

Murphy, K. P. (2012). *Machine learning: a probabilistic perspective*. MIT press.

Nakai, T., Koide-Majima, N., and Nishimoto, S. (2021). Correspondence of categorical and feature-based representations of music in the human brain. *Brain and Behavior*, 11(1):e01936.

Nakai, T., Koide-Majima, N., and Nishimoto, S. (2022). Music genre neuroimaging dataset. *Data in Brief*, 40:107675.

Penny, W. D., Friston, K. J., Ashburner, J. T., Kiebel, S. J., and Nichols, T. E. (2011). *Statistical parametric mapping: the analysis of functional brain images*. Elsevier.

Slaney, M. et al. (1993). An efficient implementation of the patterson-holdsworth auditory filter bank. *Apple Computer, Perception Group, Tech. Rep*, 35(8).

Smith, S. M., Jenkinson, M., Woolrich, M. W., Beckmann, C. F., Behrens, T. E., Johansen-Berg, H., Bannister, P. R., De Luca, M., Drobnjak, I., Flitney, D. E., et al. (2004). Advances in functional and structural mr image analysis and implementation as fsl. *Neuroimage*, 23:S208–S219.

Synigal, S. R., Teoh, E. S., and Lalor, E. C. (2020). Including measures of high gamma power can improve the decoding of natural speech from eeg. *Frontiers in human neuroscience*, 14:130.

Tipping, M. E. (2001). Sparse bayesian learning and the relevance vector machine. *Journal of machine learning research*, 1(Jun):211–244.

Tzanetakis, G. and Cook, P. (2002). Musical genre classification of audio signals. *IEEE Transactions on speech and audio processing*, 10(5):293–302.

Waskom, M. L. (2021). Seaborn: statistical data visualization. *Journal of Open Source Software*, 6(60):3021.

Zue, V. W. and Seneff, S. (1996). Transcription and alignment of the timit database. In *Recent Research Towards Advanced Man-Machine Interface Through Spoken Language*, pages 515–525. Elsevier.
